# Predicting the Effect of RSW Parameters on the Shear Force and Nugget Diameter of Similar and Dissimilar Joints Using Machine Learning Algorithms and Multilayer Perceptron

**DOI:** 10.3390/ma17246250

**Published:** 2024-12-20

**Authors:** Marwan T. Mezher, Alejandro Pereira, Tomasz Trzepieciński

**Affiliations:** 1 Departamento de Deseño na Enxeñaría, Universidade de Vigo, 36310 Vigo, Spain; apereira@uvigo.es; 2 Institute of Applied Arts, Middle Technical University, Baghdad 10074, Iraq; 3 Department of Manufacturing Processes and Production Engineering, Rzeszow University of Technology, Al. Powst. Warszawy 8, 35-959 Rzeszow, Poland; tomtrz@prz.edu.pl

**Keywords:** resistance spot welding, machine learning, artificial neural network, shear force, nugget diameter, relative importance (RI), SHAP

## Abstract

Resistance spot-welded joints are crucial parts in contemporary manufacturing technology due to their ubiquitous use in the automobile industry. The necessity of improving manufacturing efficiency and quality at an affordable cost requires deep knowledge of the resistance spot welding (RSW) process and the development of artificial neural network (ANN)- and machine learning (ML)-based modelling techniques, apt for providing essential tools for design, planning, and incorporation in the welding process. Tensile shear force and nugget diameter are the most crucial outputs for evaluating the quality of a resistance spot-welded specimen. This study uses ML and ANN models to predict shear force and nugget diameter responses to RSW parameters. The RSW analysis was executed on similar and dissimilar AISI 304 and grade 2 titanium alloy joints with equal and unequal thicknesses. The input parameters included welding current, pressure, welding duration, squeezing time, holding time, pulse welding, and sheet thickness. Linear regression, Decision tree, Support vector machine (SVM), Random forest (RF), Gradient-boosting, CatBoost, K-Nearest Neighbour (KNN), Ridge, Lasso, and ElasticNet machine learning algorithms, along with two different structures of Multilayer Perceptron, were utilized for studying the impact of the RSW parameters on the shear force and nugget diameter. Different validation metrics were applied to assess each model’s quality. Two equations were developed to determine the shear force and nugget diameter based on the investigation parameters. The current research also presents a prediction of the Relative Importance (RI) of RSW factors. Shear force and nugget diameter predictions were examined using SHapley (SHAP) Additive Explanations for the first time in the RSW field. Trainbr as the training function and Logsig as the transfer function delivered the best ANN model for predicting shear force in a one-output structure. Trainrp with Tansig made the most accurate predictions for nugget diameter in a one-output structure and for shear force and diameter in a two-output structure. Depending on validation metrics, the Random forest model outperformed the other ML algorithms in predicting shear force or nugget diameter in a one-output model, while the Decision tree model gave the best prediction using a two-output structure. Linear regression made the worst ML predictions for shear force, while ElasticNet made the worst nugget diameter forecasts in a one-output model. However, in two-output models, Lasso made the worst predictions.

## 1. Introduction

Welding is the predominant method used for connecting metallic materials among many kinds of joining techniques. It is widely used in many industrial sectors such as railroads, civil construction, automobiles, and shipbuilding owing to its favourable attributes such as impermeability, robust joints, and quick joint formation [1]. The Resistance Spot Welding (RSW) process is a widely used welding procedure in the transportation and aerospace industries owing to its advanced level of automation [2,3]. As a cost-effective method for joining metal sheets of diverse types and thicknesses, resistance spot welding (RSW) has seen widespread application across several industries. Nevertheless, the quality of the weld is directly impacted by a number of production variables, including the following: welding current, time of welding, squeezing and holding, electrode degradation, and sheet misalignment [4,5]. Minimising the potential of spot-welding failure necessitates an extremely reliable RSW quality checking technique. Nowadays, destructive testing procedures like peel, chisel, or tensile-shear tests are often used by vehicle manufacturers to attest to the quality of RSW, which is typically determined by the nugget diameter and welding strength.

Due to their low density and outstanding strength, titanium and its alloys find uses in industrial disciplines including chemical engineering, aviation, and the medical industry [6,7]. Their unique mechanical and physical qualities also make them useful in these areas. Welding techniques such as electron beam welding, tungsten inert gas (TIG) welding, and laser welding are used to join titanium alloys [8,9]. However, these methods are not without their flaws, such as the fact that laser welding processes are expensive to set up and run [6,7,8,9,10,11]. Also, the TIG process produces a coarse weld structure. There are two main problems with disregarding the friction stir technique for welding titanium sheets: the high melting point of the titanium alloy and the contact pressure of the metal sheets with the friction tool [12,13,14].

For the next generation of automobile components, grades of stainless steel have been explored owing to their greater corrosion resistance and impact-absorption capability than mild steels [15,16,17]. The transportation network of modern cities could not function without metro vehicles and intercity fast trains. Due to its low cost, robust characteristics, high corrosion resistance, and excellent weldability in the RSW process, austenitic stainless steel finds significant application in the production of components for metro vehicles [18,19,20,21,22]. The traditional arc welding technique is not appropriate for welding austenitic stainless steel (ASS) because of its high thermal expansion coefficient, in contrast to mild steels and aluminium alloys. In light of this, the RSW process has taken into account the best welding technique for producing the ASS metro vehicle parts [23,24].

Automotive and aerospace industries could benefit from composites including titanium alloy and stainless steel. The qualities of the dissimilar metals ensure that they are both strong and lightweight, which is exactly what is needed [25,26,27]. An encouraging approach to lowering spacecraft mass might be to partially replace steel components with titanium and titanium alloy [28].

Neglecting welding quality is a frequent source of accidents, which endangers both industrial output and individual safety [29]. The proliferation of related safety issues is directly proportional to the surging demand for valid tools for estimating weld quality. It is common practice in engineering to use ANNs and other machine learning (ML) models to aid manufacturing, as they are well-suited to handling nonlinear issues [30]. Researchers in the field have predicted weld quality using ANN and ML.

Using the characteristics of the voltage signal as input to the neural network (NN) model, Wan et al. [31] were able to accurately forecast the spot weld strength and nugget size. Chang et al. [32] used a backpropagation (BP) neural network to forecast the leg length of asymmetric fillet root welds, achieving an error rate of less than 7%. With the goal of identifying the location and quality of the automobile body’s resistance spot welds, Dai et al. [5] suggest a deep learning-based network model for miniature object recognition. Geng et al. [33] proposed a machine learning model using the generative adversarial network (GAN) for diagnosing weld nugget defects and subsequently enhancing weld quality. Subsequently, the model used convolutional neural networks (CNN) to accurately classify the various types of weldment defects. The results demonstrated that the model achieved an accuracy of over 94% in diagnosing weld nugget defects. Machine learning algorithms like ensemble Gradient boosting Regressor (GBR), Extreme Gradient boosting Regressor (XGBR), and Categorical Boosting Regressor (CBR) were used by Kumar et al. [34] to estimate the shear strength of AA6082/AA5083 friction-stirred specimens. With an R^2^ of 0.94 and an RMSE of 10.51, respectively, they showed that XGBR performed better than the other models. The predictive abilities of various AI/ML models with interpretation utilising techniques such as SHAPley Additive exPlanations (SHAP), feature significance, and feature interactions have been analysed by Naser [35]. Results showed that XGBoost outperformed both deep neural networks and Light Gradient Boost.

Based on NN observations, Arunchai et al. [36] employed ANN for RSW to forecast weld quality. A total of 95% of the predictions made by the model were accurate. An ANN was implemented by Martin et al. [37] to evaluate the impact of AISI 304’s RSW characteristics on the material’s pitting corrosion behaviour. Managing overfitting during training and precisely selecting hidden layer neurones are essential for accurate prediction, as demonstrated by their findings. Siamakmanesh et al. [38] worked on the analysis of grain size and hardness for friction-stirred Ti joints adopting ANN models, and the outcomes showed that the mean absolute error (MAE) dropped to 4.77%. A number of ML models, including Support vector machine (SVM), ANN, Decision tree (DT), Random forest (RF), Naïve Bayes (NB), and K-Nearest neighbour (KNN), were used by Srivastava et al. [39] to forecast the position of corrosion fatigue cracks in ship structural weldments. The DT and RF models outperformed the other four ML models, with an accuracy of 99%. Panza et al. [40] developed an ANN model by inputting the parameters of electrode displacement data. This model was used to estimate the contact area between the electrode and the sheet, as well as predicting the wear of the electrode.

Using an online monitoring system that was informed by signals produced during welding, Chen et al. [41] evaluated the quality of the titanium RSW joints’ assessment. In separate research, Chen et al. [42] introduced a backpropagation neural network model for predicting the quality of automobile welds; they found an R^2^ value ranging from 0.81 to 0.96. Kershaw et al. [43] adopted a data-driven multilayer perceptron model for forecasting the inter-diffusion layer thickness and expulsions during the RSW process. Xia et al. [44] presented a novel online method for evaluating weld expulsion in resistance spot welding utilising a newly built multi-sensor monitoring system. This method relies on the instantaneous aspects of process data, including dynamic resistance, voltage, and electrode displacement. By combining the artificial neural networks and the multi-objective genetic algorithm (GA), Pashazadeh et al. [45] were able to find the ideal variables of welding current, welding time, and pressure. Furthermore, they were able to achieve the highest joint strength using the genetic algorithm and the ANN model fitness function. In the same way, Hamidinejad et al. [46] specified the RSW parameters and the joint strength. In their study, Mezher et al. [47,48,49] applied ANN studies with the aid of experimental investigation to test RSW joint quality. Szwajka et al. [27] evaluated the mechanical performance of Ti6Al4V and DP600 steel RSW joints. Barrak et al. [50] studied the mechanical properties of RSW joints for AISI 1005. Abdullah et al. [51] used a rivet die in order to extrude AA 5052 into pre-cut AISI 1006.

The analysis of RSW joint quality is dependent on the simultaneous interaction of many parameters like weld current; weld, holding, and squeezing times; pulse welding and pressure. Assessing the impact of a single parameter on joint quality irrespective of other factors is a challenging task in practice. Hence, sophisticated deep learning methods, analytical modelling, analysis of variance, ANNs, machine learning, genetic algorithms, and so on are used. Machine learning algorithms and artificial neural networks (ANNs) are the most popular and well-understood of the approaches listed. The number of experiment dataset samples and the implemented ML algorithms determine the effectiveness of machine learning models. Nevertheless, a variety of factors affect the performance of ANN models, such as the number of hidden layers [52], the training algorithm [53], the number of neurons in the hidden layers [54], the quality of the input data [55], the neuron activation function [56], the case dataset size, and the output numbers, whether they are one or multiple.

The current literature is lacking in research into the impact of RSW process variables on joint quality standards, specifically the shear force and nugget diameter of similar and dissimilar welded samples from AISI 304 and grade 2 Ti alloy based on the ANN and machine learning models. In the present study, the responses of shear force and nugget diameter were examined based on the RSW variables, including welding current, pressure, welding time, holding time, squeeze time, and pulse welding. Also, no paper has been found to study the effect of unequal sheet thickness on shear force and nugget diameter, and the absence of mathematical equations for the shear force and nugget diameter has motivated the authors to introduce the current work. Moreover, as a novelty to the RSW field, the Relative importance (RI) of the RSW parameters was predicted for the first time by adopting various machine learning algorithms.

## 2. Experimental Methodology

### 2.1. Material Properties

**Figure 1:**
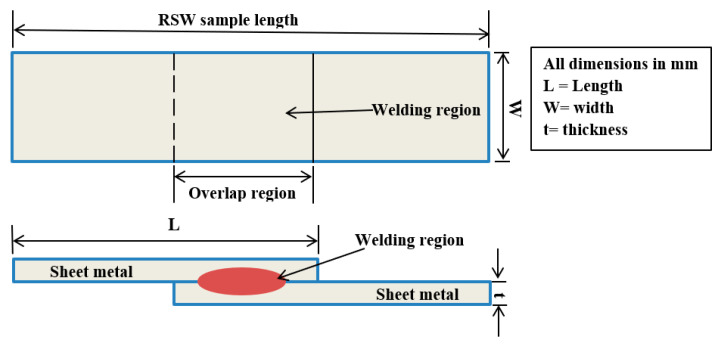
Schematic illustration of the tensile RSW sample.

In this section, the material properties of the welded sheet are presented. In this study, austenitic stainless steel 304 and titanium alloy (grade 2) with thicknesses of 0.5 and 1 mm were used to make similar and dissimilar resistance spot-welded joints. The specimens’ dimensions were selected as a lap-joint assembly based on the specifications of the AWS [57] as depicted in Table 1. A lap-joint configuration of the RSW samples is schematically shown in Figure 1.

Table 2 and Table 3 present details of the mechanical properties and chemical composition, respectively, of the investigated sheet material. In order to remove and eliminate any cutting-edge burr, surface contaminants, and the oxide layer, all titanium and austenitic stainless steel samples were mechanically polished before conducting the welding experiment with grit abrasive paper No. 800.

All titanium alloy grade 2 samples were mechanically polished with No. 800 grit abrasive paper to remove cutting edge burr, surface contaminants, and the oxide layer that had developed before being subjected to the RSW process. Once the surface was cleaned, any remaining impurities were removed using ethanol. This pre-cleaning is critical in RSW to prevent the occurrence of expulsion welds, which may happen at random on contaminated surfaces.

The RSW machine (SUNARC SA company, Esparreguera, Barcelona, Spain) set used a dome-shaped electrode. It was made of a chromium-zirconium-copper alloy that was in group A, class 2, type B. It had a contact area of 4 mm diameter. Pneumatic supply was employed to provide the pressure. Welding current, welding pressure, welding, squeezing and holding times, and pulse welding were utilized as the RSW process parameters and programmed into the welding machine via the digital screen attached to the machine. The experiments were executed at standard laboratory room temperature.

The present study included 250 RSW samples; this was achieved by partitioning the welding cases into 10 cases, with 25 samples in each case. Specimens of RSW were distributed as follows: 75 samples for welding equal titanium sheets, 25 samples for welding sheets with an equal thickness of 1 mm (coded with the letter A), 25 samples for welding sheets with an equal thickness of 0.5 mm (coded with the letter B), and 25 samples for welding sheets with unequal thicknesses of 0.5 and 1 mm (marked with the letter C). In the same way, there were 75 samples for welding similar AISI 304 sheet thicknesses of 0.5 and 1 mm, and coded with D, G, and H letters. The dissimilar RSW samples between AISI 304 and grade 2 Ti alloy, each consisting of 100 samples, were marked as E, F, I, and J cases, each containing 25 specimens. The E case refers to an equal thickness of 1 mm of both sheet metals, and case J represents an equal thickness of 0.5 mm. Case F, on the other hand, represents welded samples with an unequal thickness of 0.5 mm for Ti and 1 mm for AISI 304, and case I refers to an unequal thickness of 1 mm for Ti and 0.5 mm for AISI 304.

**Figure 2:**
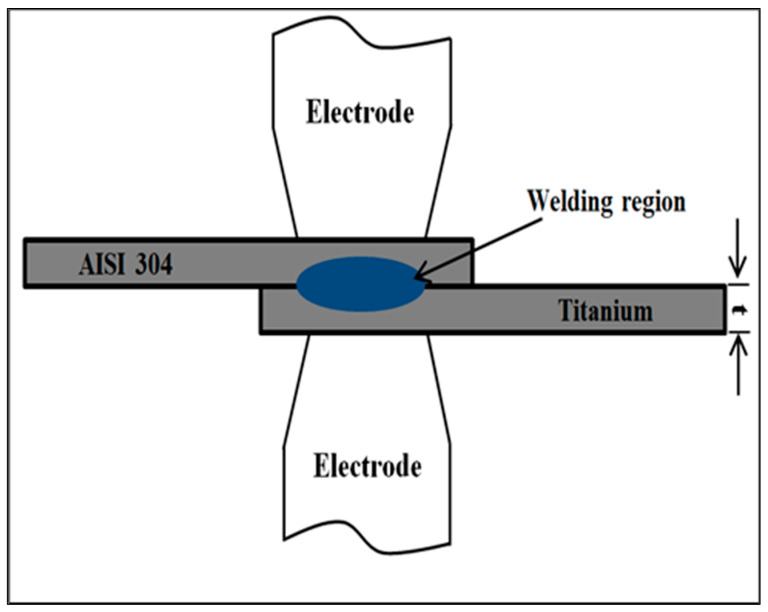
Schematic illustration of RSW process.

**Figure 3:**
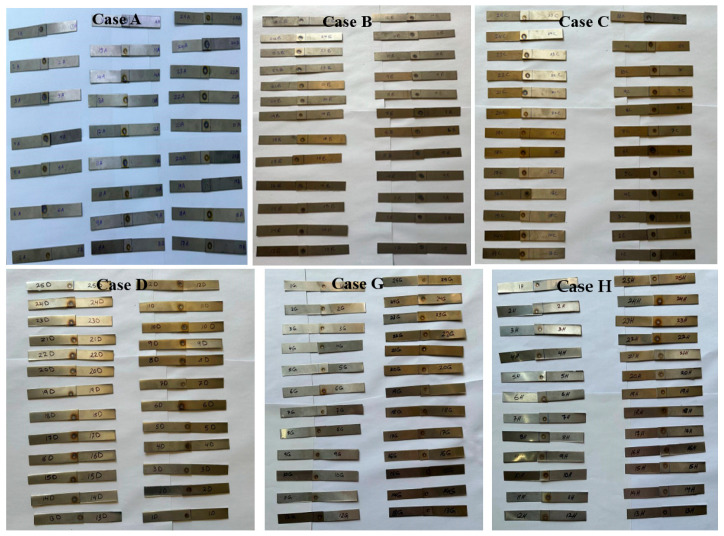

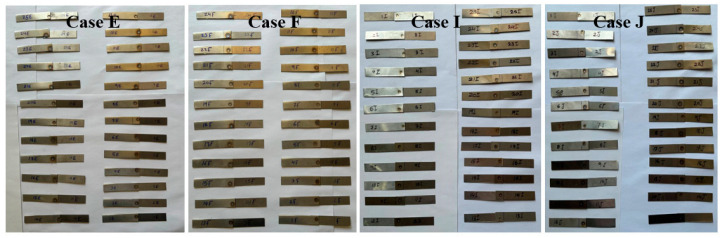
Resistance spot-welded specimens of the ten cases (**A**–**J**).

The welding parameters comprise a welding current range of 5000–7000 A, a range of 0.6–1.4 s for both welding and squeezing times, a holding time range of 0.5–1.5 s, a pulse welding range of 1–5, and a pressure range of 2–8 bar. Before commencing the welding process, an X was placed on the top face centre of the samples to facilitate accurate positioning of the electrode tip within the overlap zone. Figure 2 depicts a simplified schematic of the experimental welding apparatus. The Design of the Experiment (DOE) follows the Taguchi technique using Minitab 19.0 software to organize testing for the ten RSW scenarios [47,48,49], as shown in Table 4. Figure 3 shows resistance spot welded samples of all ten cases.

### 2.2. Welding Quality Characterization

**Figure 4:**
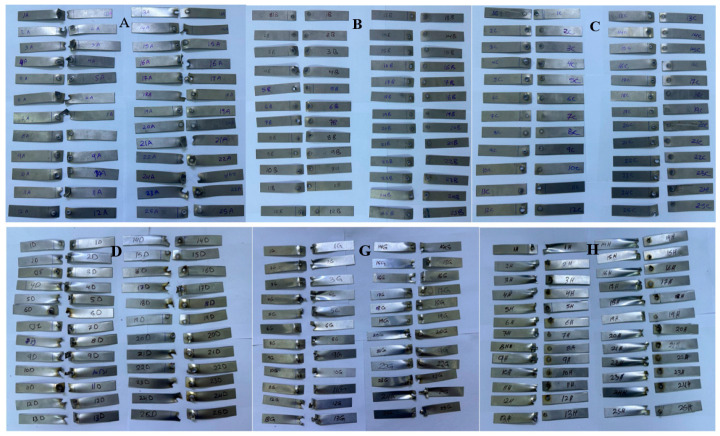

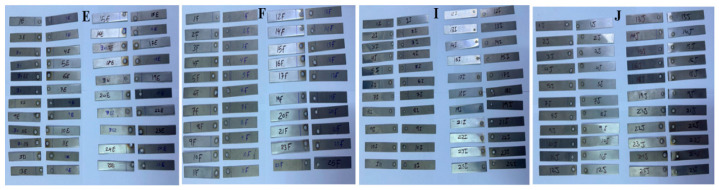
Fractured RSW samples of the ten cases (**A**–**J**).

The mechanical performance of the RSW joints was assessed using tensile shear force measurements. In all ten cases, the RSW samples were subjected to tensile shear force to determine the peak load necessary to break the RSW samples. At room temperature, the cross-head speed was 5 mm/min, and the maximum load was 100 kN. This universal testing equipment (Laryee Technology Co., Ltd., Beijing, China.) was used for the tensile shear tests. Extracting the force displacement curve from the tensile shear test allowed us to determine the peak load after specimen breakage. In addition, measuring the nugget diameter allowed us to assess the final spot diameter in accordance with the ANSI/AWS/SAE requirements [57]. When it comes to resistance spot weld quality, the nugget diameter is key. Figure 4 shows the samples that broke during the tensile shear test for the ten cases.

## 3. Application of Predictive Models

Experiments are crucial for evaluating the welding quality of RSW joints; however, they are costly and time-consuming. Additionally, the rapid expansion of computing power has enabled the simulation of real-world RSW processes through computer technologies based on machine learning (ML) and artificial neural network (ANN) models. Due to their robust adaptive learning and nonlinear mapping capabilities, various ML and ANN algorithms have also emerged as reliable instruments for investigating the nonlinear relationships between multi-input parameters and target responses. Therefore, various machine learning algorithms like linear regression, Decision tree, Support vector machine (SVM), Random forest (RF), Gradient boosting, CatBoost, K-Nearest neighbour, Ridge, Lasso, and ElasticNet, along with two different structures of Multilayer Perceptron ANNs, were adopted for studying the effect of the RSW variables on welding quality in terms of shear force and nugget diameter. The GridSearchCV method was used for the first time in the RSW field to optimize the hyperparameters for all ML models, ensuring better model performance. Also, a pipeline technique was used to handle data preprocessing, feature selection, and model training in a structured way. This improves data readability and maintainability. It combines all the steps (data cleaning, feature selection, and model training) into one cohesive pipeline. For advanced preprocessing, the author used a ColumnTransformer to distinguish between numerical and categorical variables. Categorical features are encoded with OneHotEncoder. Numerical features are scaled using StandardScaler.

### 3.1. Linear Regression ML Algorithm

Linear regression is a popular machine learning method used for predictive analysis, predicting continuous or quantitative variables like sales, salary, age, and product price. It connects a dependent variable (goal) (y) to one or more independent variables (predicted variables) (x). In this study, inputs are independent and output is dependent, examining how the dependent variable changes in response to changes in the independent variable [58]. This algorithm is mathematically represented as in Equation (1):

Y = b_0_ + b_1_.X_1_ + b_2_.X_2_ +b_n_.X_n_ ….. + ε (1)

where:

Y is the dependent (target) variable;
b_0_ is the intercept of the line;
b_1_ is the linear regression coefficient;
X is the independent (predicted) variable;
ε is the error term.

**Figure 5:**
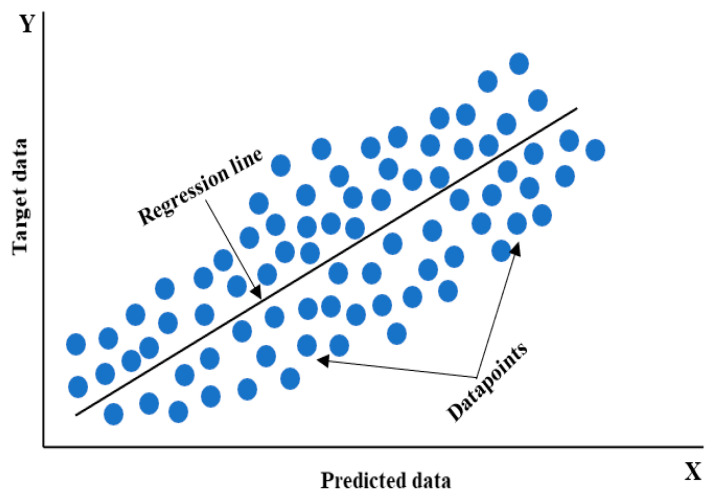
Schematic of the linear regression curve.

The linear regression model depicts a straight line with a certain slope that represents the correlation between the variables, as shown in Figure 5.

Simple linear regression uses a single independent (target) variable to predict a numerically dependent (predicted) variable. This study predicts the shear force or nugget diameter as one output variable using this approach. Second, multiple linear regression predicts the dependent variable using several independent variables (targets). This holds true when making predictions for both the shear force and nugget diameter simultaneously.

### 3.2. Decision Tree ML Algorithm

**Figure 6:**
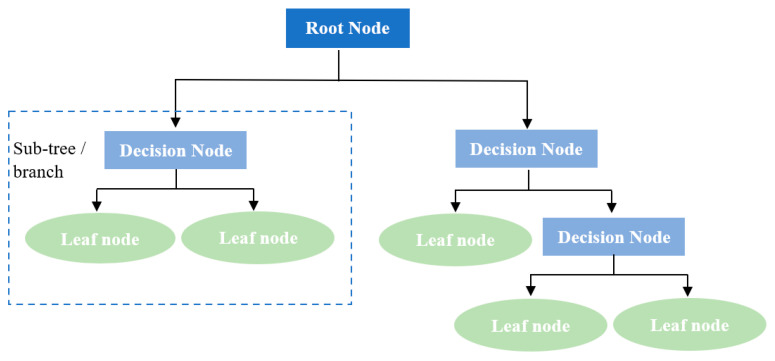
Schematic of the Decision tree model.

A Decision tree is a supervised machine learning algorithm that handles both classification and regression issues. However, it primarily addresses classification problems. A tree organizes the classifier, with core nodes reflecting dataset attributes, branches representing decision rules, and leaf nodes expressing results. A decision tree has two sorts of nodes: decision and leaf. Decision nodes can make any choice and have multiple branches, whereas leaf nodes cannot. Tests and judgments employ dataset features. The term “decision tree” is used because, like a tree, it begins with a root node and then grows more branches, forming a structure that resembles a tree, as portrayed in Figure 6.

The Decision Tree ML model starts at the root and partitions the dataset into related subgroups. Leaf nodes are the final output nodes, and splitting divides the decision node into sub-nodes based on criteria. A tree grows from the main tree, pruning unwanted branches. The top node is the parent node, and the rest are child nodes. When using a decision tree to predict a dataset’s class, the process starts at the root node, compares attribute values with sub-nodes, and progresses. The procedure continues to the tree’s leaf node. The hyperparameters used for the Decision tree model were [5, 10, 20, None], and the minimum samples split were [2, 5, 10] in order to find the optimal hyperparameters.

### 3.3. Support Vector Machine (SVM) ML Algorithm

Support vector machine (SVM) is a regression algorithm developed by Vapnik and colleagues for supervised machine learning. Parrella [59] created the SVM family’s Support Vector Regression (SVR) toolkit to solve non-linear problems between the training dataset’s input and output. SVR builds a hyperplane that captures a specific percentage of the data within a margin of error, unlike traditional regression methods that minimize disparities between predicted and actual values. Vapnik’s theory [60] defines the hyperplane as depicted in Equation (2).

w^T^. u + b = 0 (2)

The hyperplane was constructed to meet the following inequality conditions:

w^T^. u + b ≥ +1y_i_ = +1 (3)

w^T^. u + b ≤ +1y_i_ = −1 (4)

From Equations (2)–(4), the following equation is derived:

y_i_ (w^T^. u + b) ≥ +1   I = 1, 2, 3, ….., N (5)

where:

w^T^: weight vector transposed version;
u: the sample data;
b: the bias.

**Figure 7:**
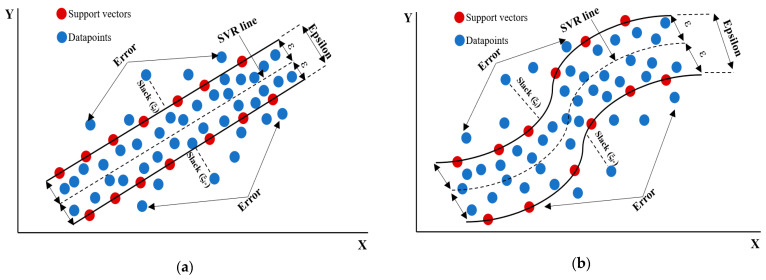
Diagrams of the SVR ML model: (**a**) linear SVR, (**b**) non-linear SVR.

Support vector regression (SVR) is a method that forecasts a continuous target variable by finding a function that maximizes the difference between actual and predicted values. It reduces prediction errors by fitting the line inside a margin. SVR uses data points closest to the regression line to calculate margins as support vectors. There are two types of SVR: linear separability, where a straight line separates a dataset into categories, and non-linear SVR, which often works with non-linear data. Real-world data, including welding operations, is not linearly separable, hence non-linear SVR are used. Pre-processing techniques convert training data into a higher-dimensional feature space, but this increases overfitting and computing complexity. The “kernel trick” simplifies processing by replacing dot product computations with kernel functions. SVR can characterize non-linear correlations between input features and target variables using linear, polynomial, and radial basis functions [61,62]. The diagram in Figure 7 represents the SVR concept with the two types.

SVR reduces complexity without compromising accuracy in forecasting target variables by creating an ε-epsilon tube around the hyperplane, allowing variance between actual and anticipated values to balance model complexity and generalization. SVR can be mathematically formulated as follows:

*f*(*x*) = w^T^.ϕ(x) + b (6)

where:

f(*x*): SVR function;
w^T^: weight vector transposed version;
ϕ(x): the kernel function;
b: the bias.

The aim is to find a flat-aligned function (x) that maintains maximum divergence of ε from training data goals, reducing sensitivity to input data fluctuations and overfitting. Linear functions with low coefficient ω are flat. Optimization difficulties often include errors. Data points outside the ε-insensitive tube as indicated by Equation (7) are treated as slack variables (ξ_i_ and ξ_i_*) [62]. A regularization parameter C > 0 balances total deviations beyond the ε-insensitive tube and model complexity. The SVR model was trained with the following hyperparameters for the purpose of calculating the optimum parameters: C: [0.1, 1, 10] and kernel: [linear and rbf].

(7) $$\min f\left(w,\;b)=0.5\;w^{T}.\;w+C\;\sum_{i=1}^{m}(\xi i+\xi i^{*}\right)$$

Equation (3) is subject to the following conditions:

y_i_ − w^T^. ϕ(x) − b ≥ ε + ξ_i_ (8)

w^T^. ϕ(x) + b − y_i_ ≤ ε + ξ_i_^*^ (9)

ξ_i_, ξ_i_* ≥ 0

The data above ε will represent ξ_i_, while ξ_i_* represents the data under ε.

### 3.4. K-Nearest Neighbour (KNN) ML Algorithm

**Figure 8:**
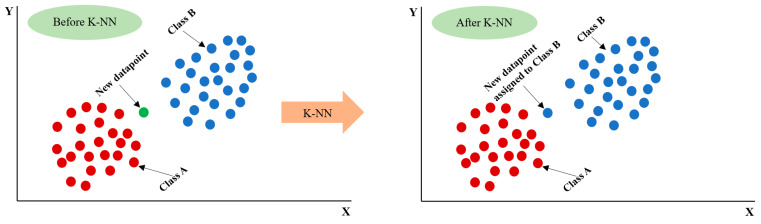
Diagram of the KNN ML model.

The K-Nearest Neighbour (KNN) algorithm is a supervised machine learning technique used to address classification and regression issues [63]. This algorithm was developed by Evelyn Fix and Joseph Hodges in 1951, and then enhanced by Thomas Cover. It is used in pattern recognition, data mining, and intrusion detection. The algorithm classifies new data based on similarities with existing data, assigning it to the category with the highest degree of similarity. It is non-parametric and applicable to real-world situations, relying on no data distribution assumptions. The KNN algorithm saves the dataset during training and classifies new data into a similar category. The following diagrams in Figure 8 show how the KNN algorithm works.

The application of cross-validation enhances model performance by randomly dividing training and test data into n subsets. Repeatedly, each subset is used for training and testing. The average cross-validation error measures performance. Hyperparameters are optimized using an exhaustive grid search approach, which checks all combinations to find the best. KNN uses metric, weight, and n-neighbour hyperparameters. KNN uses distance metric to classify data by proximity, using hyperparameters like Minkowski, Euclidean, and Manhattan. To predict the new dependent variable Y, KNN analysed the K closest samples around Y, using Euclidean distance to measure data point proximity. Similar findings were expected from multidimensional space, with similar results. KNN uses the idea that nearby data points may help predict the dependent variable, ensuring similar results for data points with identical multidimensional properties. The hyperparameters for the KNN model were n_neighbours: [3,5,10] for identifying the best parameters. The following is a description of N samples along with the distance from X and Y variables to N samples.

(X_1_, Y_1_) ….. (X_N_, Y_N_) (10)

d(X, Y) was the distance from the Kth sample.

d(X_1_, Y_1_) ≤ …. ≤ d(X_N_, Y_N_) (11)

Selected *K*th samples were calculated to determine the sample average.

(12) $$ý==\sum_{i=1}^{k}\frac{Y(i)\;}{K}$$

### 3.5. CatBoost ML Algorithm

CatBoost is an open-source, high-performance Gradient Boosting algorithm for categorical data [64]. It handles categorical data, eliminating the need for pre-processing methods like one-hot or label encoding [65]. CatBoost uses target-based encoding and statistical methods to reduce overfitting, making it effective with smaller datasets. It works well with default settings, eliminating the need for hyperparameter tuning [66]. CatBoost generates 1000 six-level binary two-leaf trees by default. Automatic learning is the most efficient, as training dataset features and iteration count limit automated learning rate computation. Raising the learning rate and reducing iterations accelerates training. The CatBoost model was trained with the following hyperparameters for obtaining the optimized parameters: model depth: [6, 8, 10], learning rate: [0.01, 0.1, 0.2], and model iterations: [100, 200].

### 3.6. Gradient-Boosting ML Algorithm

**Figure 9:**
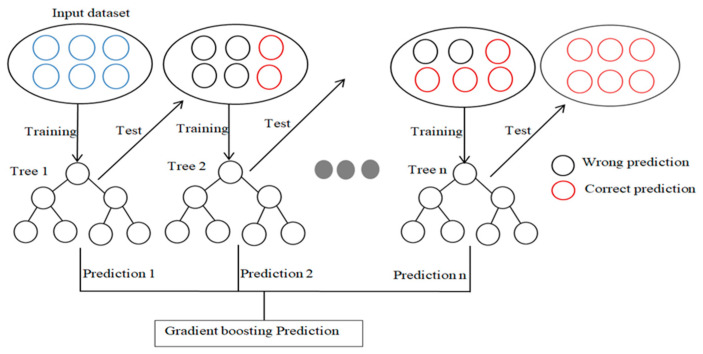
Diagram of the Gradient-boosting ML algorithm.

Gradient-boosting decision trees are a popular machine learning method for mapping functions using known input and target variables. They handle complex connections, are computationally simple, and have greater interpretability [67,68]. This method is practical for implementation and produces real-world predictions [69,70]. It serves as both a regressor and classifier, and is used in embedded failure prediction algorithms. In this study, it was used to predict the shear force and nugget diameter of welded joints with a depth of 6 and a minimum sample division of 2, 1000 trees and least-squares loss. Figure 9 illustrates Gradient boosting. The least squares loss reduces error by appending prediction points to the fitted line. Gradient boosting enhances weak learners by learning from and improving on previous models. The term “gradient” comes from gradient descent, which minimizes loss function. The boosting approach involves iterative learning nodes, fitting results to the dataset, analysing errors, and learning previously learned nodes until a specified number of iterations is reached. The hyperparameters for the Gradient-boosting model were n_estimators: [50, 100, 200] and learning rate: [0.01, 0.1, 0.2] in order to determine the optimum parameters.

### 3.7. Random Forest (RF) ML Algorithm

**Figure 10:**
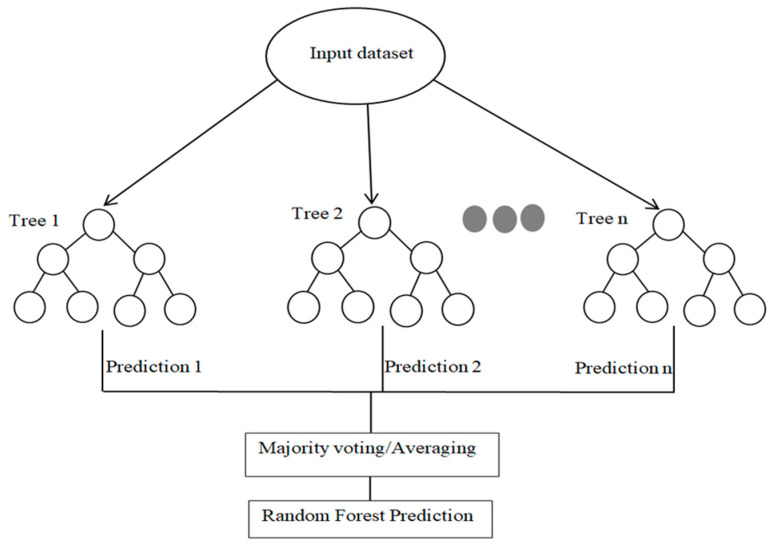
Diagram of the Random forest ML algorithm.

Random forest (RF) is a machine learning algorithm that trains multiple decision trees simultaneously and extracts their mode (classification) or mean prediction (regression). Launched in 2001 by Leo Breiman and Adele Cutler, it offers exact prediction, high-dimensional data processing, and contribution- and relevance-based input prioritization. The RF model can be useful for classification and regression, using bagging to build decision trees on each training data subset, reducing variance and over-fitting. Adjusting setup settings improves the algorithm’s predictive power. The setup settings for a decision tree in machine learning involve a minimum number of data points at each leaf node to prevent overfitting [71,72]. The decision tree randomly selects a subset of characteristics at each split, resulting in less association and more predictability. Hyper-parameters also influence model effectiveness, algorithm pattern recognition, and analysis speed [73]. Dataset hyper-parameter tuning requires cross-validation for optimal prediction performance. The model employed 10-fold cross-validation. Data was divided into 10 subgroups, and one was chosen to test the trained model in each iteration [74,75]. The hyperparameters for the Random forest model were n_estimators: [50, 100, 200] and maximum depth: [none, 10, 20] for obtaining the ideal parameters. The illustration scheme for the Random forest employed in the present investigation is illustrated in Figure 10.

### 3.8. Lasso Regression ML Algorithm

L1 regularization, or Lasso Regression, is a technique used for regression analysis to reduce overfitting by reducing coefficients to zero. This technique is particularly useful for avoiding unnecessary data characteristics and highlighting minimally influenced aspects. It helps in enhancing linear regression by fitting data points to a line, minimizing the sum of squared discrepancies between observed and predicted values, as shown in Equation (13).

(13) $$\min\;\mathrm{RSS}=\sum {\left(yi-ýi\right)}^{2}$$

where:

*yi*: the observed value;
*ýi*: the predicted value.

This algorithm assists with real-world datasets with multicollinearity by regularizing them with a penalty term to prevent overfitting. It balances model complexity and accuracy, promoting sparse solutions with zero coefficients. It also aids in feature selection by automatically identifying and eliminating irrelevant variables. To do this, it multiplies the coefficients of the predictors (β_i_) by the regularisation parameter (λ), after which these are added to the residual sum of squares (RSS), as shown in Equation (14). The degree of regularisation is controlled by this regularisation parameter.

(14) $$\mathrm{RSS}+\lambda \;\times \;\sum \left|\beta_{i}\right|$$

where:

β_i_: the coefficients of the predictors;
λ: the tuning parameter.

Selecting the tuning parameter (λ) is crucial in lasso regression. Typically, cross-validation techniques are used to ascertain the optimal lambda value that achieves a balance between predictive accuracy and model complexity. To determine the optimum hyperparameters for the Lasso model, the current study employed the following hyperparameters: alpha: [0.01, 0.1, 1].

### 3.9. Ridge Regression ML Algorithm

Ridge Regression, also known as L2 regularization, is an extension of linear regression that uses regularization, like the lasso method. It shifts coefficients toward zero but never to absolute zero, and feature weights never approach zero. When a coefficient is zero, the model eliminates a predictor. However, it retains regression coefficients, making it unsuitable for feature selection. This is one of the limitations of ridge regression. Another issue is that ridge regression cannot discriminate between predictors at high multicollinearity levels. Aiming to identify the optimized hyperparameters for the Ridge model, this model was trained using alpha set to [0.1, 1, 10].

### 3.10. ElasticNet Regression ML Algorithm

Regularized ElasticNet regression is a combination of lasso and ridge regression, designed to handle multicollinearity and large predictors. It includes lasso and ridge L1 and L2 penalty terms, which may improve stability and performance. ElasticNet is reliable for varied regression circumstances, as it uses L1 and L2 penalties for feature selection and regularization. The hyperparameters for the ElasticNet model were alpha: [0.01, 0.1, 1] and L1 ratio: [0.2, 0.5, 0.8] for calculating the ideal parameters.

### 3.11. Artificial Neural Network (ANN) Modeling

It was McCulloch and Pitts’ work in the 1940s that laid the groundwork for the concept of neural networks. Their primary assumption was that neural networks were capable of solving any logic or mathematical equation. Neural networks (NNs) have lately attracted the attention of tens of thousands of scholars from all disciplines. There is no scientific field that does not have some kind of relationship with neural networks. Numerous industries have adopted ANNs, including healthcare, aerospace, military, the arts, film, music, and many more. Rather than depending on assumptions when building their model, neural networks improve their performance via data learning. Many individuals believe that neural networks are only oversimplified representations of how the brain really handles neural inputs [76,77].

Training a model to correctly anticipate process dynamics using input and output data is one of many applications of the neural network approach [78,79]. This method works particularly well in welding processes, since it is quite difficult to have a thorough knowledge of the physical mechanics. Learning new skills is possible for the neural network because of training data. The procedure begins with the input variables and concludes with a comparison of the predicted and actual outputs. Next, the network modifies the value of the layer-to-layer links. The process is terminated by the network after it produces favourable results on the training set. Another approach to improve network convergence is to evaluate the network using validation data that does not include the training set.

#### 3.11.1. Multilayer Perceptron (MLP) Neural Network

The structure, connections, inputs, and outputs of a network define its topology. An ANN’s topology may be defined by its input and output layer numbers, neuron counts within each layer, and the transfer functions that connect them [79]. Input, output, and hidden layers are the standard components of an ANN’s design. Several neurons constitute each layer of the network. There are the same number of neurons in the input layer as there are input variables, and the same number of neurons in the output layer for each input. Due to the transfer function [80], the neurons in the layers are able to transfer weight data. In order to train an ANN model using a multilayer perceptron (MLP) architecture, this study used a back-propagation learning approach. The MLP is defined as follows by Equation (15):

(15) $$y=f\left(net\right)=\left(\sum_{i=1}^{n}w_{i}x+b\right)$$

where:

y: the outputs;
f: the activation function;
x: the input;
w_i_: the weights;
b: the bias.

**Figure 11:**
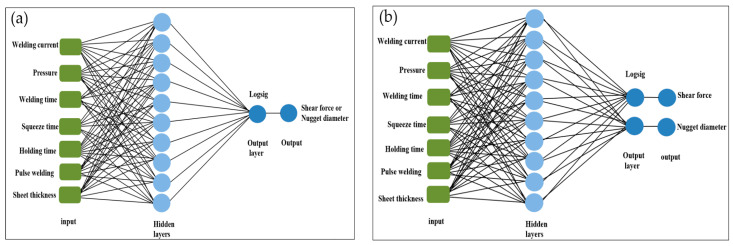
Neural network structure of the RSW process, (**a**) one output, (**b**) two outputs.

For the purpose of predicting the tensile shear forces and nugget diameter of the ten RSW scenarios, two MLP structures were constructed using MATLAB R2021a. Actual measured data from the welded samples were used to create the inputs and targets dataset using the RSW technique. A total of seven neurons contribute to the inputs, which include welding current, pressure, welding time, squeeze time, holding time, pulse, and sheet thickness. Figure 11 shows the schematic for the net structures used in this study. Each of these structures has one hidden layer with ten neurons linked to the input and output layers. In addition to a learning rate of 0.01 and a performance target of 0.001, the other primary training parameter used in this work was 1000 epochs. In order to determine the optimal model and structure, it is important to highlight that several training and transfer functions were tested and trained.

#### 3.11.2. Transfer and Training Functions

During training, the goal is to optimise the network’s parameters such that the resulting prediction map is as accurate as possible. All of the many optimization methods have a common name, and training functions are just one of them. Training functions are algorithms that are used to train networks in how to identify and react to inputs. The trained data set, biases, weights, and performance objective are among the several parameters that decide the training function. Improving the network’s ability to generate fast, accurate predictions requires defining a suitable training function. In order to convert inputs into outputs for this study, thirteen separate training functions were executed in MLP networks. Table 5 provides a comprehensive description of the training functions that were used in this work.

When using ANN, the inputs to each node are multiplied by their respective weights, and then the total of these products is calculated. The sum is then transferred via a transfer function, which is a mathematical operation. Each layer’s output is determined by the total weights that enter that layer, after which a transfer function processes the collected weights. Among other factors, the network topology is a major factor in the complicated process of establishing suitable transfer functions. Improving prediction accuracy was the goal of this research, which included using several transfer functions. Three functions were considered in the scope of this study: the pure linear function (purelin), the log sigmoid transfer function (logsig), and the hyperbolic tangent sigmoid function (tansig). The transfer functions may be determined via Equations (16)–(18). The logsig transfer function was consistently employed for the output layer in all cases.

(16) $$Tansig\left(n\right)=\frac{2}{1+e^{-2n}}-1$$

(17) $$Logsig\left(n\right)=\frac{1}{1+e^{-n}}$$

(18) $$Purelin\left(n\right)=n$$

### 3.12. Data Distribution

The results of the RSW joints, like shear force and nugget diameter, may be used as targets for predicting the welded joint outcomes. Data must be partitioned in order to construct training, validation, and test datasets. The efficiency of any model might be drastically altered by dividing a dataset into a training set and a testing set. According to Shahin et al. [81], the correlation between the ratios of various subsets of data is not readily apparent. The dividing ratio is a major challenge with datasets, as stated by Zhang et al. [82,83], and there is currently no generic solution to this issue. The data allocation in this research was 80% for training, and 20% for testing. The training dataset was partitioned into validation and test subsets to guarantee that the model learnt and evaluated all data samples. Therefore, the training dataset, which included 80% of the whole dataset, was partitioned as follows: 70% for training, 15% for validation, and 15% for testing. It should be mentioned that the testing dataset (20%), which was kept for final testing reasons, was not included in the training datasets. Data for training, testing, and validation were derived from real data obtained from 500 experimental samples related to the RSW joints.

### 3.13. Evaluation Criteria

Examining the accuracy of a prediction model depends on selecting suitable assessment criteria; however, using actual data is essential to improving the performance of the model. This study evaluated and quantified the degree of agreement between the actual and projected results by comparing and validating several training and transfer algorithms. The evaluation of results and the reduction of mistakes is further complicated by the necessity of selecting an acceptable validation standard. All of the ANN models that were trained and evaluated in this study were compared using suitable criteria in order to assess their performance. The metrics used in the analysis were mean squared error (MSE), root mean squared error (RMSE), mean error (ME), mean absolute error (MAE), mean relative error (MRE), and the coefficient of determination (R^2^).

Minimal error is indicated by low values of MSE, RMSE, and MAE, which are approximately 0. A high R^2^ value, which is near to 1, indicates exceptional performance. Under these scenarios, a reliable and steady performance may be ensured. Conversely, a notable disparity between the RMSE and MAE values suggests major inconsistencies in the error distribution. Furthermore, when contrasted with other validation methods, MSE, RMSE, and MAE provide a more exact evaluation. Both MSE, RMSE, and MAE provide stability, although MSE and RMSE are more error-sensitive. The error (*E*), MSE, RMSE, ME, MAE, MRE, and R^2^ may be computed using the following formulae:

(19) $$E=(y_{p}-y_{t})$$

(20) $$MSE=\frac{1}{N}\sum_{i=1}^{N}(y_{p}-y_{t}{)}^{2}\;\;\mathrm{or}\;\;MSE=\frac{1}{N}\sum_{i=1}^{N}(E{)}^{2}$$

(21) $$RMSE=\sqrt{MSE}$$

(22) $$ME=\frac{1}{N}\sum_{i=1}^{N}(y_{p}-y_{t})\;\;\mathrm{or}\;\;ME=\frac{1}{N}\sum_{i=1}^{N}(E)$$

(23) $$MAE=\frac{1}{N}\sum_{i=1}^{N}(|y_{p}-y_{t}|)\;\;\mathrm{or}\;\;MAE=\frac{1}{N}\sum_{i=1}^{N}(|E|)$$

where:

*y_p_*: the predicted value;
*y_t_*: the target value;
*N*: total number of experiments.

### 3.14. Evaluation of the RSW Parameters Contribution

Analysis of the relative or feature importance examines how the welding input parameters affect the outputs. However, the research shows how each factor is weighted in the predictive model through an examination of how the prediction averages vary as the significance value of a feature fluctuates. Substituting input variables with lower feature important values for those with higher feature importance values drastically changes the findings. The Random forest, decision tree, gradient-boosting, and CatBoost algorithms already include a built-in tool for relative importance. Based on the literature review, it seems that no publication has examined the relative importance of similar and dissimilar welded joints created by the RSW technique.

Another criterion which was applied to interpret the relative importance of the inputs is the SHAP (SHapley Additive exPlanations), for the first time in the field of resistance spot welding. Lundberg suggested SHapley Additive Explanations (SHAP) as a way to explain machine learning model predictions [84]. Machine learning models use the SHAP approach to elucidate their outputs. The methodology is based on Shapley values, a principle from cooperative game theory, that facilitates an equal distribution of each feature’s contribution to the predicted output. The SHAP calculation approach involves iterating over all potential feature subsets and assessing the variance in prediction outcomes for each subset to ascertain the Shapley value for each feature. Equation (24) [85] calculates the SHAP value.

(24) $$SHAP\;value(\Phi )\sum_{S\subseteq N\backslash \{i\}}\frac{\left|S\right|!(\left|N\right|-\left|S\right|-1)!}{|N|!}\left[f\left(S\cup \left\{i\right\}\right)-f(S)\right]$$

where:

N: set of all input features;
S: set of non-zero feature indices;
S⊆N∖{i}: subset of all features excluding feature {i};
ƒ(S): the model’s prediction when only the features in subset S are present;
ƒ(S ∪ {i}): the model’s prediction when the feature i is added to the model.

## 4. Results and Discussion

### 4.1. One-Output Structure

**Figure 12:**
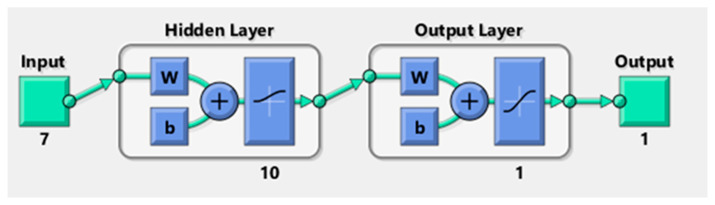
ANN model with one-output structure.

**Figure 13:**
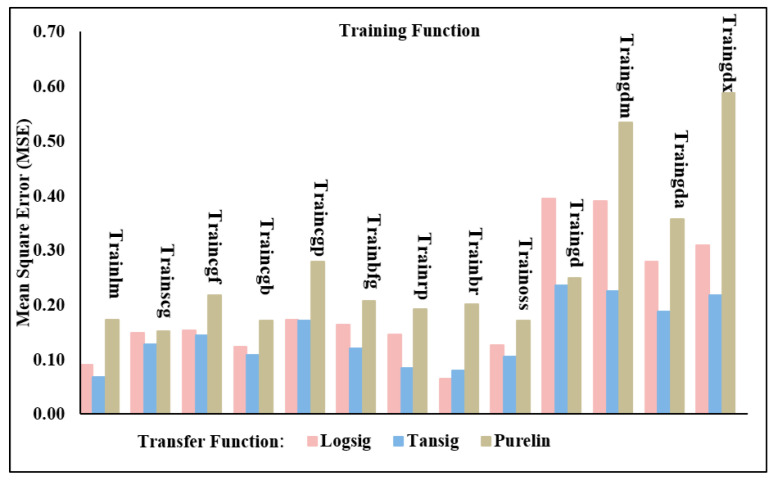
MSE of shear force using various training and transfer functions.

Using a one-output neural network topology, the neural network models’ performance was assessed in this phase. In other words, several models of artificial neural networks, trained with varying transfer and training functions, were used to predict either the shear force or the nugget diameter as shown in Figure 12. Table 6 and Table 7 show the actual experimental results of the shear force and nugget diameter, respectively. The evaluation metrics utilized for checking the accuracy of the ANN shear force model of the resistance spot-welded joints with a single-output neural network structure are detailed in Table 8 and Figure 13. According to the validation metrics, the model performs best when R^2^ approaches 1, and MSE, RMSE, MAE, and MRE approach zero. This means that the suggested model performs better than others.

Based on the data presented in Table 8 and Figure 13, it is evident that the Trainbr with Logsig model produced the most accurate predictions for the shear force. The MSE, RMSE, and MAE values were 0.06487, 0.254687, and 0.153859, respectively. On the other hand, using Purelin to evaluate Trainbr reduced these metrics to 0.20094, 0.448265, and 0.351234 for MSE, RMSE, and MAE, respectively. The worst outcomes of a one-output structure in forecasting shear force occurred when Traingdx used the Purelin transfer function, yielding MSE, RMSE, and MAE values of 0.58793, 0.766763, and 0.621736, respectively.

**Figure 14:**
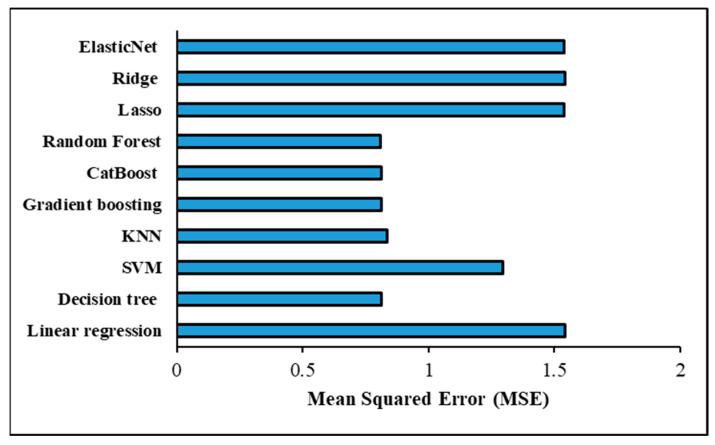
MSE of shear force using different ML models.

**Figure 15:**
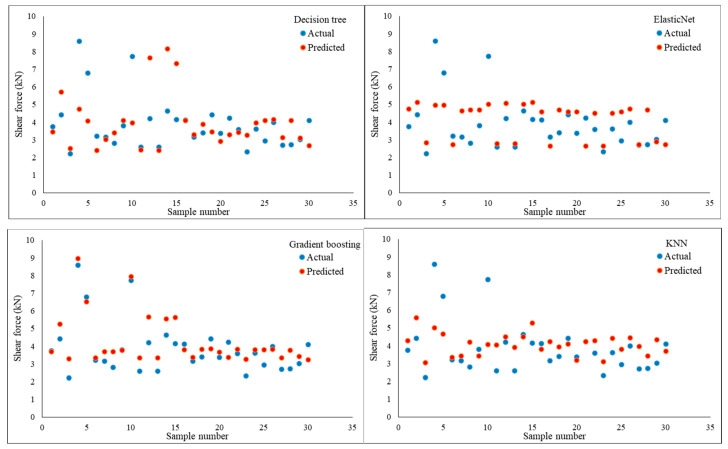

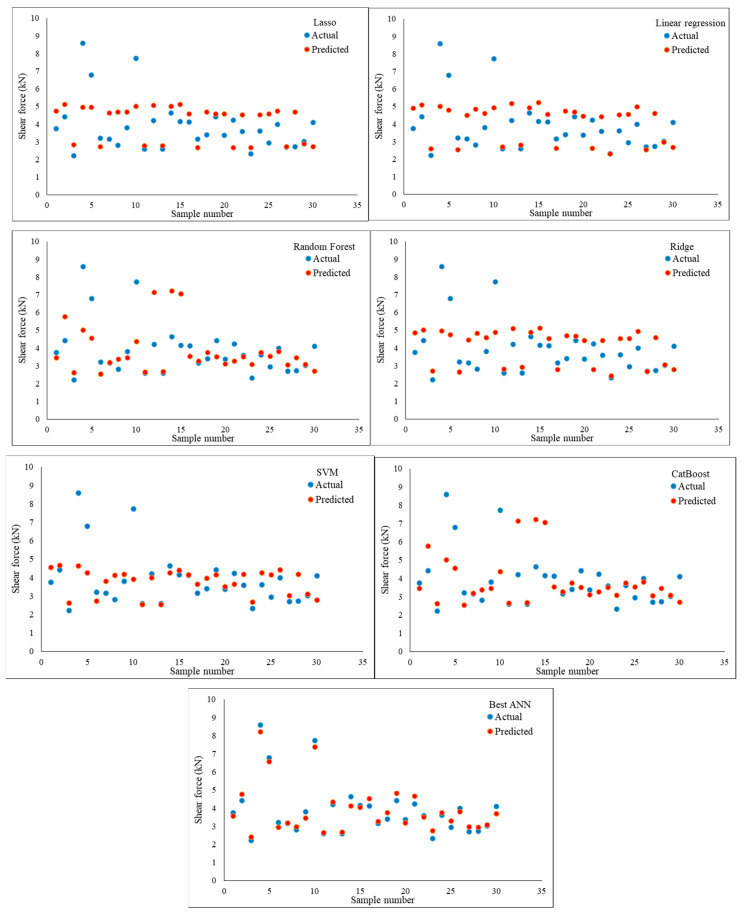
Actual and predicted shear force using different ML models.

The assessment criteria used to verify the ML models’ accuracy in predicting the shear force in a one-output structure are shown in Table 9 and Figure 14 under the optimised hyperparameters using the GridSearchCV method. With an MSE of 0.810029 and an RMSE of 0.900016, the Random forest model was determined to have the most accurate prediction based on these measures. Models such as decision tree, CatBoost, and gradient-boosting were able to obtain assessment metrics that were almost identical, placing them on the second level of accuracy performance for prediction. For decision tree, the MSE and RMSE were 0.811960 and 0.901088; for CatBoost, they were 0.811960 and 0.901088; and for gradient-boosting, they were 0.811496 and 0.900831. On the other hand, the worst prediction findings were scored with the linear regression model, wherein MSE and RMSE were 1.543320 and 1.242304, respectively. Despite recording poor forecasting results, the Lasso, Ridge, and ElasticNet models outperformed the linear regression model in prediction performance. The assessment metrics showed that these models performed similarly; specifically, the Lasso model had an MSE of 1.538041 and an RMSE of 1.240178, the Ridge model had an MSE of 1.543218 and an RMSE of 1.242263, and the ElasticNet model had an MSE of 1.540389 and an RMSE of 1.241124. The K-Nearest neighbour (KNN) model achieved good results with MSE and RMSE of 0.834705 and 0.913622, respectively, but still lower than the prediction performance achieved with Random forest, decision tree, CatBoost, and gradient-boosting models. The evaluation metrics obtained with the support vector machine (SVM) model were 1.293762 for MSE and 1.137437 for RMSE; however, this prediction performance is not promising. Furthermore, Figure 15 shows a significant positive correlation between the predicted and actual shear force findings of the different ML models. Table 10 provides the optimized hyperparameters for all ML models using the GridSearchCV method for predicting the shear force.

**Figure 16:**
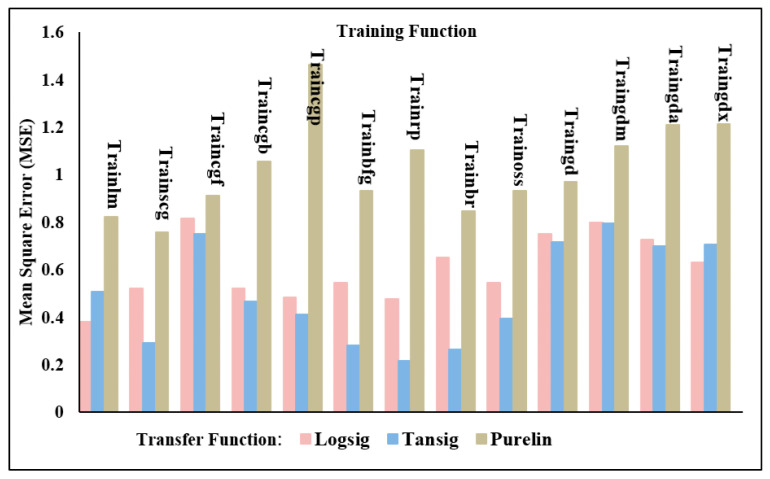
MSE of nugget diameter using various training and transfer functions.

The validation metrics used to evaluate the ANN model’s performance in predicting the nugget diameter in a one-output structure are shown in Table 11 and Figure 16. It is evident from these metrics that the most favourable prediction model was attained with Trainrp and Tansig; the resulting MSE and RMSE were 0.215283 and 0.463986, respectively. Conversely, the least accurate prediction of the nugget diameter was recorded using Purelin and the Traincgp training function; the MSE and RMSE values were 1.462530 and 1.209351, respectively. Importantly, when examined with all training functions, the Purelin transfer function produced the lowest prediction performance of the shear force or nugget diameter models in the one-output structure. Furthermore, in the one-output structure, the ANN model of the shear force achieved better validation metrics than the nugget diameter.

**Figure 17:**
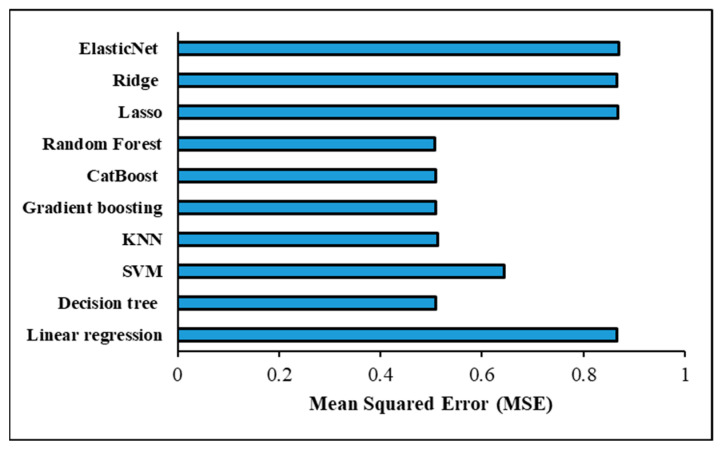
MSE of nugget diameter using different ML models.

**Figure 18:**
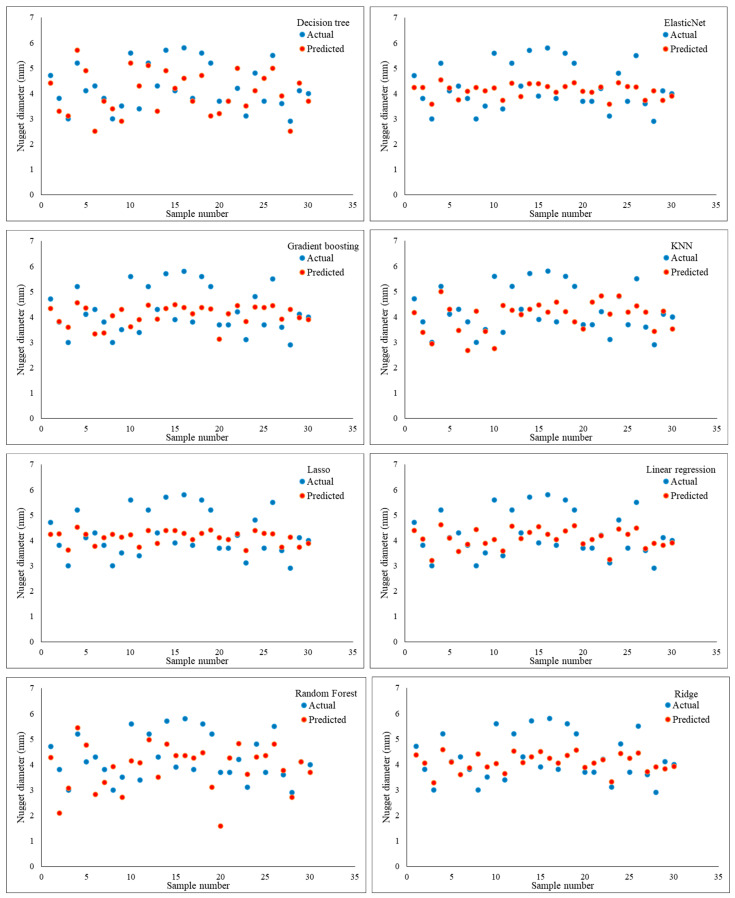

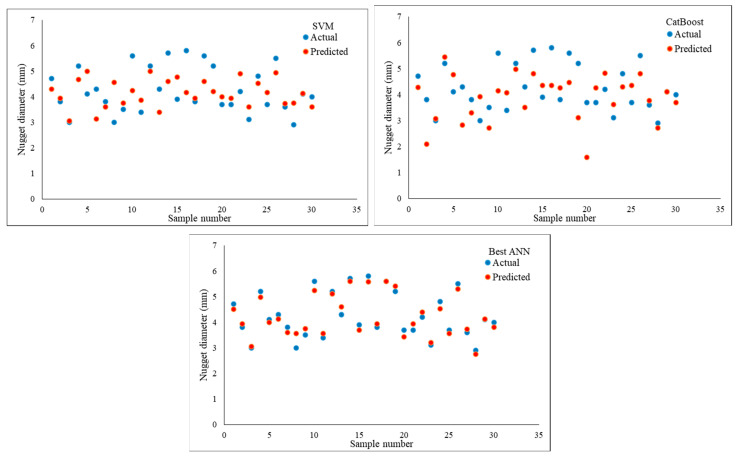
Actual and predicted nugget diameter using different ML models.

Table 12 and Figure 17 demonstrate the evaluation criteria used for confirming that the ML models accurately predicted the nugget diameter in the one-output structure. When comparing the prediction performance of the ML shear force models with that of the ML nugget diameter models, it was discovered that the nugget diameter models produced superior results. The Random forest model was found to have the highest accurate prediction with an MSE of 0.508039 and an RMSE of 0.712768. Following the trend seen with shear force models, gradient-boosting, decision tree, and CatBoost all reached second-level accuracy in prediction when tested using evaluation criteria. When it came to the Decision tree model, the MSE and RMSE were 0.509383 and 0.713711, whereas for CatBoost they were 0.509383 and 0.713711, and for Gradient boosting they were 0.508880 and 0.713358. However, the Lasso and ElasticNet models had the poorest prediction results, with MSE and RMSE values of 0.868458 and 0.931911 for the Lasso model, and 0.869719 and 0.932587 for the ElasticNet model, respectively. Despite the fact that the Linear regression and Ridge models achieved virtually the same outcomes and were slightly better compared to the Lasso and ElasticNet models, with MSE and RMSE values of 0.865809 and 0.930489 for the linear model and 0.865827 and 0.930498 for the Ridge model, respectively, this is still regarded as a poor prediction. The KNN model came in at the third prediction level, with MSE and RMSE at 0.513015 and 0.716251, respectively. The MSE and RMSE values observed with the SVM model were 0.644345 and 0.802711, respectively. Furthermore, Figure 18 shows a considerable relationship between the predicted and actual nugget diameter results of the different ML models. Table 13 provides the optimized hyperparameters for all ML models using the GridSearchCV method for predicting the nugget diameter.

### 4.2. Two-Output Structure

**Figure 19:**
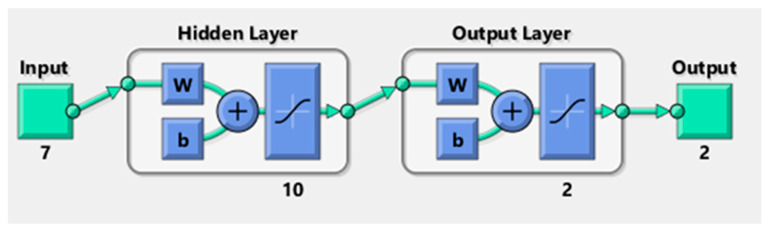
ANN model with two-output structure.

**Figure 20:**
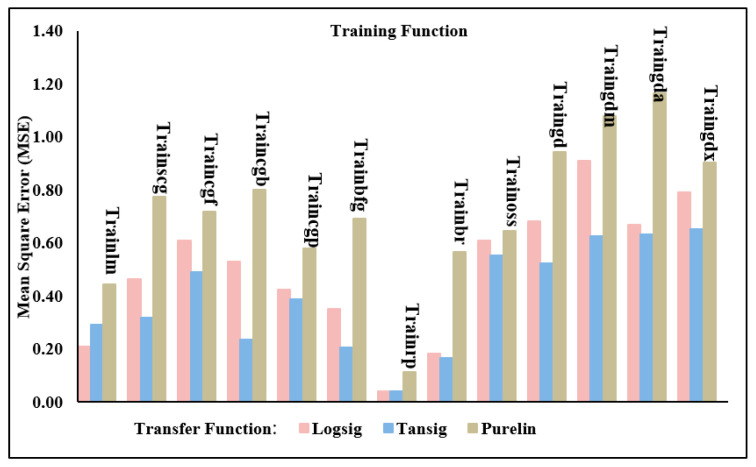
MSE of shear force and nugget diameter using various training and transfer functions.

**Figure 21:**
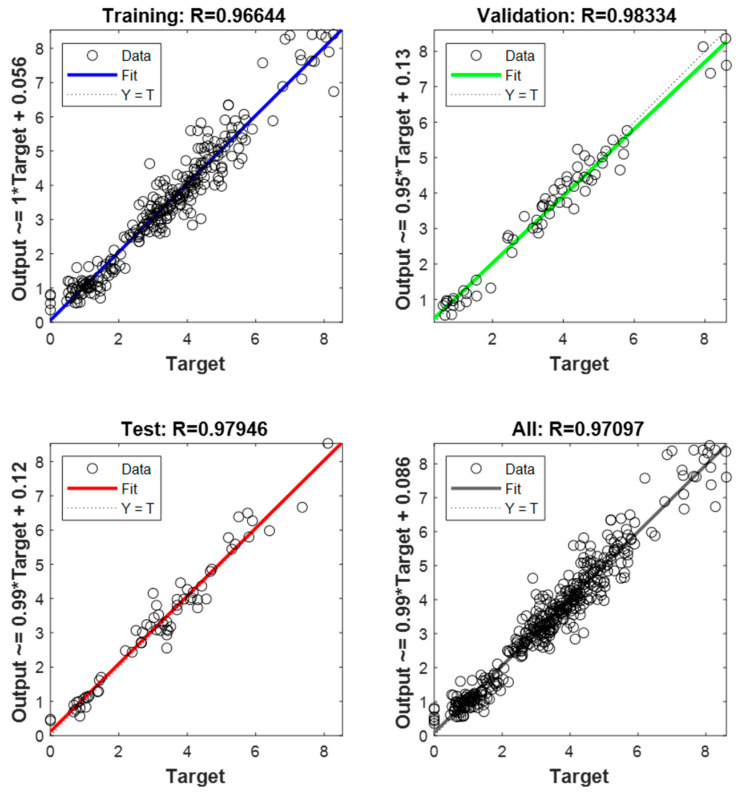
Regression curve of actual and predicted data of the best two-output ANN model using Trainrp with Tansig.

This section examines the accuracy of the findings for shear force and nugget diameter obtained by using a neural network with two outputs using a variety of neural network models. In other words, the same artificial neural network models used to forecast the shear force or nugget diameter in the one-output structure were also used to train the models for the two-output structure, as shown in Figure 19. Results from using the Trainrp training function in conjunction with the Tansig transfer function were clearly the best, as seen in Table 14 and Figure 20. Validation metrics for MSE and RMSE were 0.040719 and 0.438666, respectively. In contrast, Traingda and Purelin had the worst results, with MSE and RMSE values of 1.166745 and 1.080159, respectively. Worthy of note is that the optimal prediction model for nugget diameter in the one-output model and the two-output model for shear force and nugget diameter was attained with the same training function (Trainrp) and transfer function (Tansig). However, the predicted nugget diameter with the two-output model was more favourable than the findings in the one-output nugget diameter model. Figure 21 shows the best performance of actual and predicted data curves for the optimal two-output ANN model when using the Trainrp training function with the Logsig transfer function.

**Figure 22:**
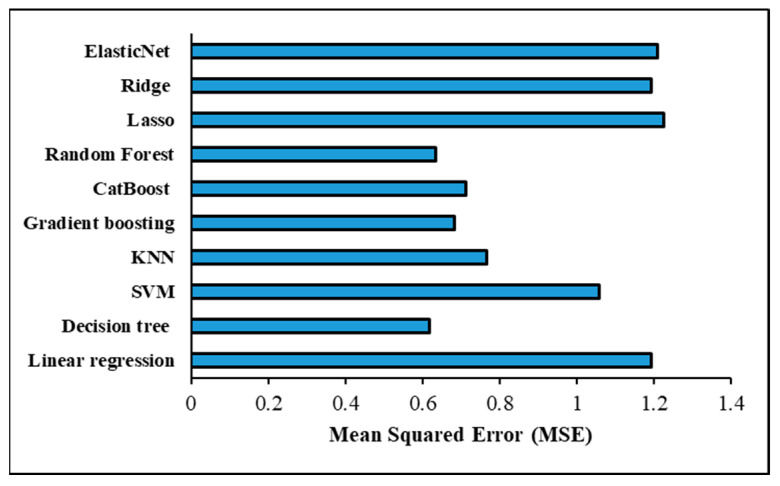
MSE of shear force and nugget diameter in the two-output structure using different ML models.

The highest prediction performance for the two-output structure was found when the Decision tree model was used, as shown in Table 15 and Figure 22, with MSE and RMSE values of 0.617400 and 0.785748, respectively. The Lasso model, in contrast, achieved the lowest prediction when evaluating the two-output model, with MSE and RMSE values of 1.226762 and 1.107593, respectively. In addition, the assessment metrics reported in Table 13 and Figure 22 were similar to those obtained by the ElasticNet, Ridge, and Linear regression models, which resulted in inferior forecasts. The second most accurate model was found when the Random forest technique was applied, with an MSE and RMSE of 0.633338 and 0.795826, respectively. Predictive models using gradient-boosting and CatBoost algorithms achieved the third level of prediction accuracy. The Gradient-boosting and CatBoost models all have different mean squared errors (MSE) and root-mean-squared errors (RMSE): 0.682354 and 0.826047, 0.711931 and 0.843760, respectively. When compared to the Gradient-boosting and CatBoost models, the KNN model’s performance was somewhat worse (MSE = 0.766689, RMSE = 0.875608). Furthermore, the SVM model produced evaluation values of 1.057539 for MSE and 1.028367 for RMSE. Remarkably, the two-structure ML model gave better results for shear force than in the one-output structure; however, the one-output structure model for nugget diameter produced the best prediction results. Table 16 provides the optimized hyperparameters for all ML models using the GridSearchCV method for predicting the shear force and nugget diameter in the two-output model structure.

### 4.3. Relative Importance (RI)

**Figure 23:**
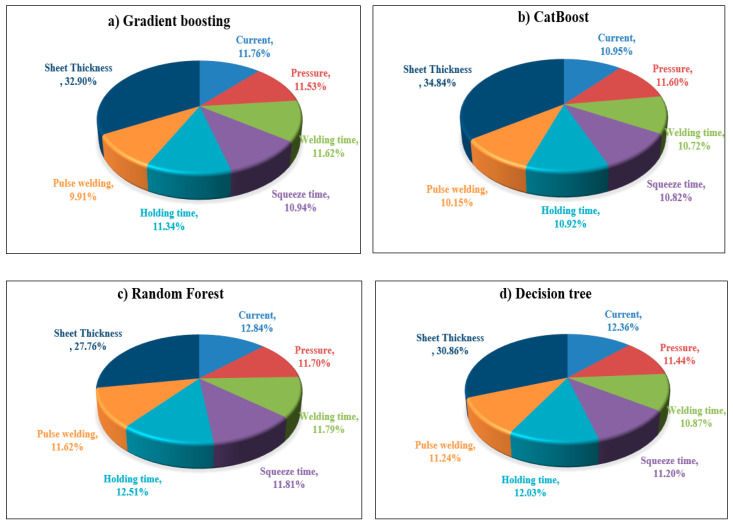
Relative importance of the RSW parameters on the shear force based on (**a**) Gradient-boosting, (**b**) CatBoost, (**c**) Random forest, (**d**) Decision tree models.

**Figure 24:**
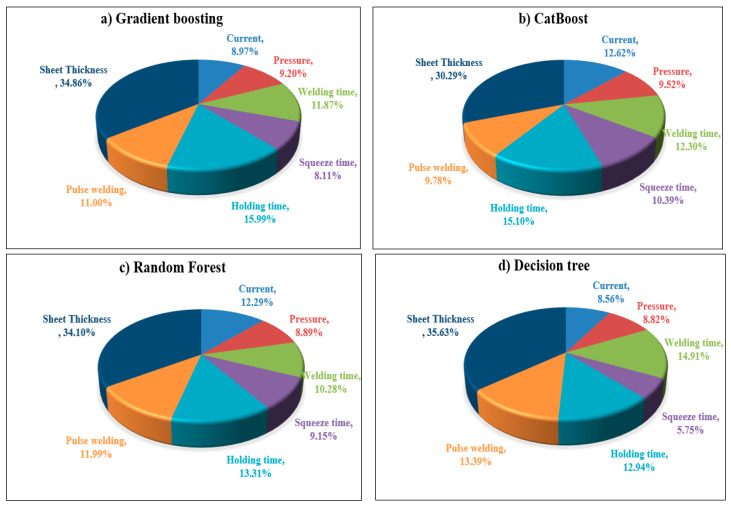
Relative importance of the RSW parameters on the nugget diameter based on (**a**) Gradient boosting, (**b**) CatBoost, (**c**) Random forest, (**d**) Decision tree models.

The effect of each RSW variable on the shear force and nugget diameter of the resistance spot-welded joints is discussed in relation to their Relative importance (RI). As seen in Figure 23 and Figure 24, several regression models, including gradient-boosting, CatBoost, random forest, and decision tree regressions, were used to evaluate the RI contribution of each parameter. Figure 23a shows that, according to the Gradient-boosting model, the sheet thickness is the most important factor affecting the shear force, with a RI of 32.90%. In contrast, pulse welding has the least influence, with an RI of 9.91%. Using the Gradient-boosting model to determine the RI with respect to the nugget diameter, as shown in Figure 24a, it was observed that the sheet thickness ranks first, with a 34.86% RI, and that pressure ranks last, with an 8.11% RI.

In the context of the CatBoost model’s relative importance analysis, Figure 23b reveals that the sheet thickness, with an RI of 34.84%, significantly influences shear force on the first level, while the pulse welding, as in the Gradient-boosting model, with a RI of 10.15%, has the least impact. Another interesting observation, based on Figure 24b, is that the sheet thickness with an RI of 30.29% is the most influential factor with respect to nugget diameter, and the pulse welding contribution of the RI was 9.52% and was recorded as the least affected factor. As with the Gradient-boosting and CatBoost models, the Random forest model shows that sheet thickness, which accounts for 27.76% and 34.10% of the RI, respectively, is the most significant component in relation to shear force and nugget diameter (Figure 23c and Figure 24c). However, with 11.62% and 8.89%, respectively, pulse welding and welding pressure had the least impact on shear force and nugget diameter. With an RI value of 30.86% and 35.63% as shown in Figure 23d and Figure 24d, the Decision tree model’s relative importance (RI) analysis showed that the biggest effects on shear force and nugget diameter always had an effect on changes in sheet thickness. Conversely, the welding time and squeeze time, which accounted for 10.87% and 5.75% RI, respectively, made the least contribution to the shear force and nugget diameter. It is noteworthy that in the context of the Gradient-boosting, Random forest, and Decision tree models, the welding current emerged as the second most significant factor affecting shear force, with RIs of 11.76%, 12.84%, and 12.36%, respectively. The CatBoost model ranked the welding pressure at the second level, exhibiting an RI of 11.60%. The second significant factor influencing nugget diameter was identified as holding time, which yielded the following RI when evaluated using gradient-boosting, CatBoost, and random forest: 15.99%, 15.10%, and 13.31%, respectively. In contrast, the Decision tree model ranked welding time second, with an RI of 14.91%.

**Figure 25:**
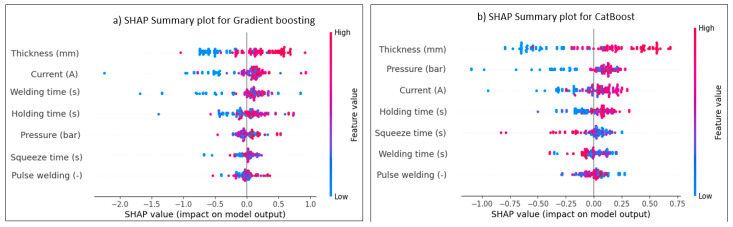

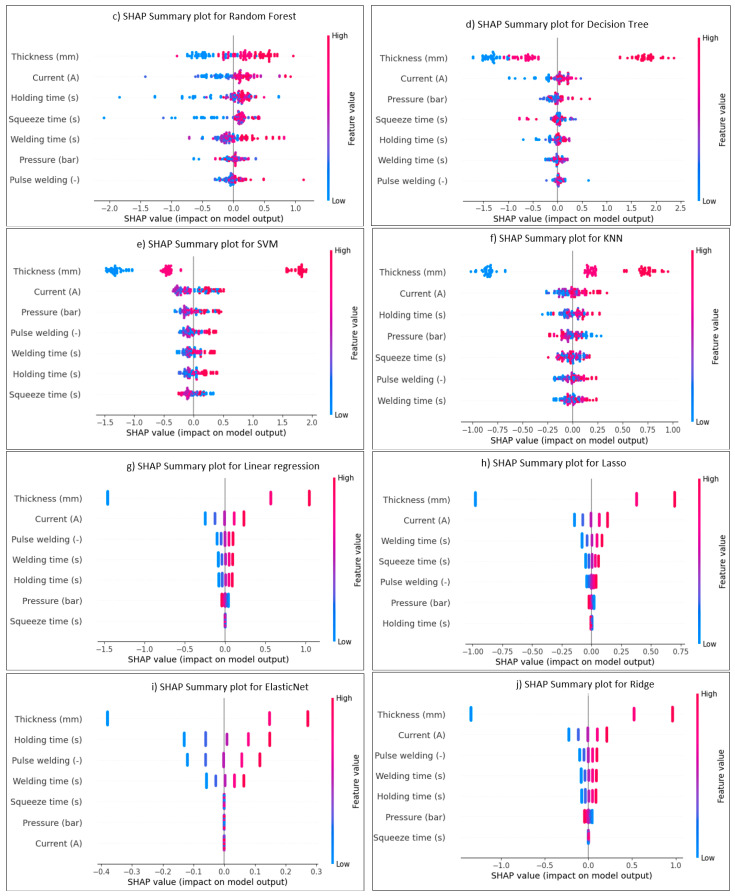
Summary plot of SHAP value impact on shear force for different algorithms: (**a**) Gradient boosting, (**b**) CatBoost, (**c**) Random forest, (**d**) Decision tree, (**e**) SVM, (**f**) KNN, (**g**) Linear regression, (**h**) Lasso, (**i**) ElasticNet, (**j**) Ridge.

**Figure 26:**
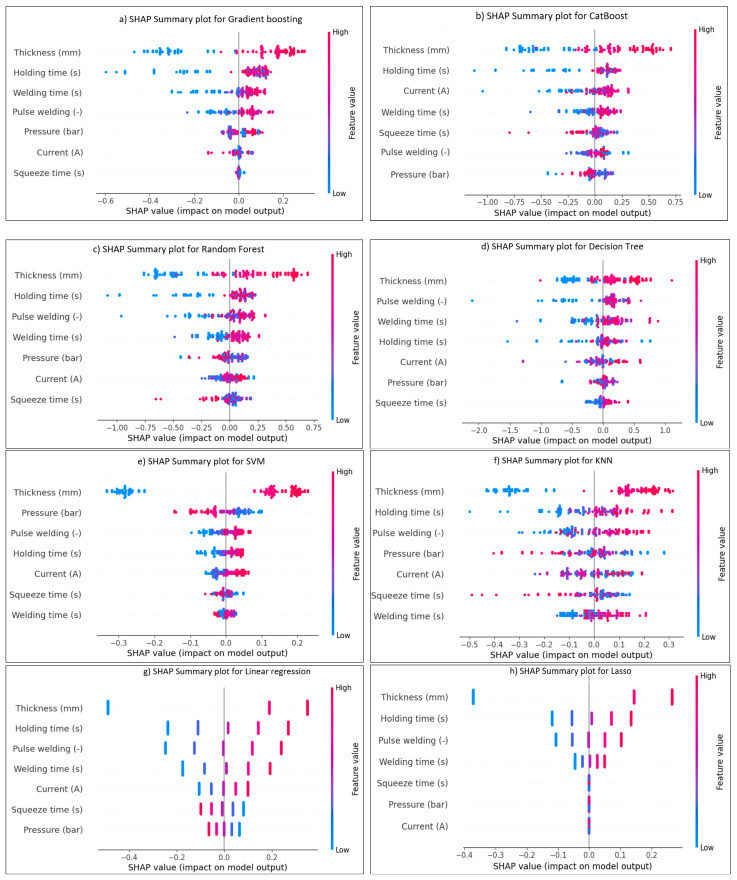

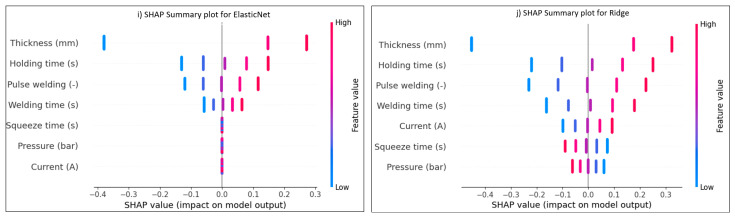
Summary plot of SHAP value impact on nugget diameter for different algorithms: (**a**) Gradient boosting, (**b**) CatBoost, (**c**) Random forest, (**d**) Decision tree, (**e**) SVM, (**f**) KNN, (**g**) Linear regression, (**h**) Lasso, (**i**) ElasticNet, (**j**) Ridge.

Model prediction performance is interpreted by the SHAP tool, which is applied to the ANN and ML models. The model’s contribution and the effect of each RSW factor may be estimated and explained. This method assists in evaluating the relative importance of each characteristic for every dataset row. Figure 25 and Figure 26 provide the summary plot of SHAP feature importance for shear force and nugget diameter of different algorithms, respectively, demonstrating the significance of each characteristic in relation to their impacts. The summary plot shows the Shapley values of the features as points. The feature’s level is defined by axis Y, while the Shapley values are shown by axis X. From least to most important, the plot colours represent the feature values. The predicted distribution of the features is shown by the Shapley values. In light of other feature values in the same row, it is important to note that identical feature values might have varying contributions to a result. In light of other feature values in the same line, it is important to note that identical feature values might have varying contributions to the prediction performance.

The colour’s values or location represent the degree of factor impact, with red values having the highest influence on the shear force and nugget diameter, and blue values having the least impact on the predicted output. The interpretation of the model is elucidated according to the positional values, determined by the locations on the right or left. A positive impact is indicated by the data on the right, whilst the values on the left indicate a negative effect. For example, the red line on the far-right side of Figure 25a represents the parameter of the sheet thickness value, suggesting that it is the most significant factor on the shear force when using the gradient-boosting algorithm, and this point is the maximum value for the sheet thickness. The furthest point in this row is situated at 1. Without this value, the predicted model of a shear force would drop below −1. Conversely, the last blue point of the welding current, positioned at −2.2, indicates that the exclusion of this value would increase the prediction by 2.2. Concerning the effect of the RSW parameters on the shear force for the other ML algorithms in Figure 25, the same interpretation used above for gradient-boosting in Figure 25a might be used for explaining the SHAP value of the RSW factors for these models mentioned in Figure 25b–j.

The effect of the SHAP value on the diameter of the nugget for various ML algorithms is shown in Figure 26. At its greatest value, for example, the red line on the far-right side of Figure 26c indicates the parameter of the sheet thickness value, which is the most influential influence on the nugget diameter when applying the random forest algorithm. On this row, 0.75 is the farthest point. The estimated model of a nugget’s diameter would fall below −0.75 in its absence. On the other hand, the last blue point of the holding time, which is located at approximately −1.15, suggests that the prediction would be increased by 1.15 if this value were excluded. This explanation regarding the SHAP value impact might apply for all other models mentioned in Figure 26. In conclusion, greater border extension across datapoints of the same feature correlates with increased effectiveness of that feature. Also, the standard deviation would vary significantly due to the convergence and divergence of data points, affecting the average error between the actual and predicted results, which in turn would impact the ML model’s performance.

**Figure 27:**
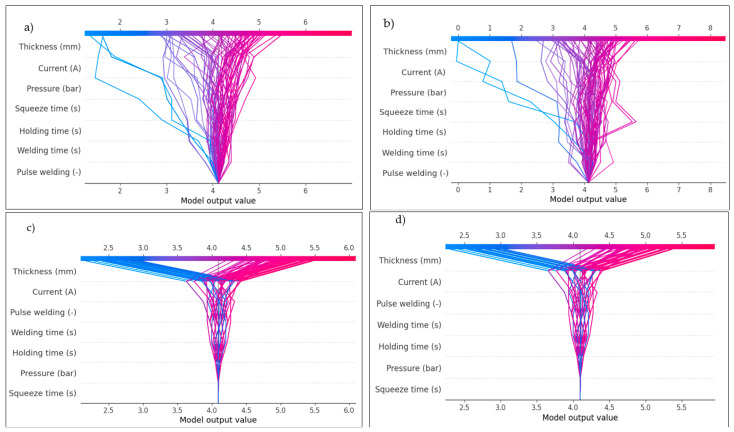
SHAP decision plot of shear force for different algorithms: (**a**) Random forest, (**b**) Decision tree, (**c**) Linear regression, (**d**) Ridge.

**Figure 28:**
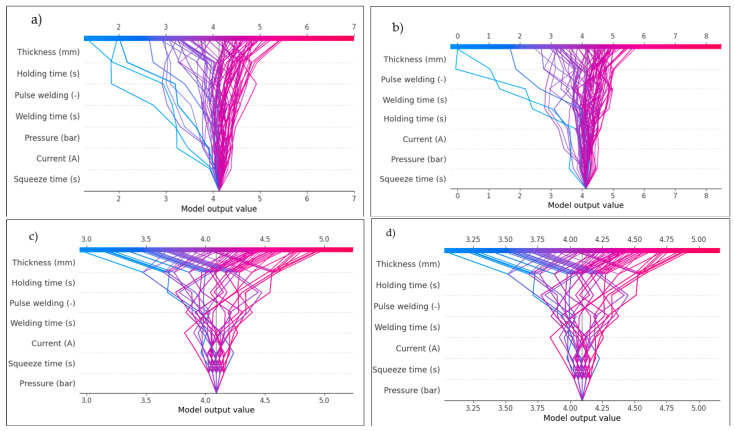
SHAP decision plot of nugget diameter for different algorithms: (**a**) Random forest, (**b**) Decision tree, (**c**) Linear regression, (**d**) Ridge.

Figure 27 and Figure 28 show the prediction models that were used to create the SHAP decision plot. This plot uses cumulative SHAP values to represent the models. Every line on the plot represents a different model prediction. The shear force and nugget diameter effect predictions are plotted in Figure 27 and Figure 28, respectively. The waterfall plot in SHAP serves as a visual instrument to elucidate the individual prediction of a model by illustrating the contribution of each feature to the final prediction. It is especially beneficial for elucidating the results of machine learning models in a manner that is both comprehensible and transparent. A model’s prediction can be seen in a waterfall plot, which shows the addition or subtraction of each feature’s SHAP value in order. It demonstrates how including each feature moves the prediction closer to the final prediction from the baseline (average prediction). For a better grasp of how each feature contributed to a single case’s prediction, this plot is essential. According to Figure 27 and Figure 28, which show waterfall plots of shear force and nugget diameter components determined under different conditions, the SHAP summary plots in Figure 25 and Figure 26 show that the order of the impacts of the RSW parameters varied across the various ML models. The estimation of parameters is thus the product of various factors, as indicated by this. For example, Figure 27a–d show that the sheet thickness is the most influential factor for all models, meaning that this is the one effective parameter that sufficiently affects the shear force, as confirmed by Figure 25. However, when comparing the least contributed factor for these models, it was shown that the Random forest and Decision tree models were less affected by the squeeze time, while the Linear regression and Ridge models were minimally impacted by the pulse welding. Concerning the waterfall plots for the nugget diameter depicted in Figure 28, it was observed that sheet thickness is the most significant variable across all models, whereas squeeze time is the least influential parameter for the Random forest and Decision tree models, and pressure for the Linear regression and Ridge models.

In SHAP, the dependency plot shows the link between a feature and the model’s output by visualizing how the value of a feature affects the SHAP values for that feature. In other words, it is useful to know how the model’s predictions shift in response to changes in the values of a single feature, considering the interactions between that feature and others. The X-axis (feature value), which represents the actual values of the feature under examination, is the primary component of the SHAP dependency plot. The Y-axis represents the SHAP value corresponding to the feature value. The SHAP value for a feature measures its contribution to the model’s output for a particular instance. Positive SHAP values indicate that the feature increases the model’s predicted value (toward a higher predicted value). Negative SHAP values indicate that the feature is reducing the model’s prediction (moving it toward a lower predicted value). Additionally, the dots on the dependent plot are color-coded according to the value of an interacting feature, often known as the interaction feature. This makes it easier to visualize the interactions between other features and the crucial feature that influences the SHAP values. Dots represent the data points in the dataset, with the dot’s location on the X-axis indicating the feature value and its position on the Y-axis indicating the SHAP value for that specific instance. It can be seen how variations in the value of the feature affect the model’s prediction for specific cases by looking at the SHAP dependency plot, which illustrates the connection between the feature and the SHAP value.

**Figure 29:**
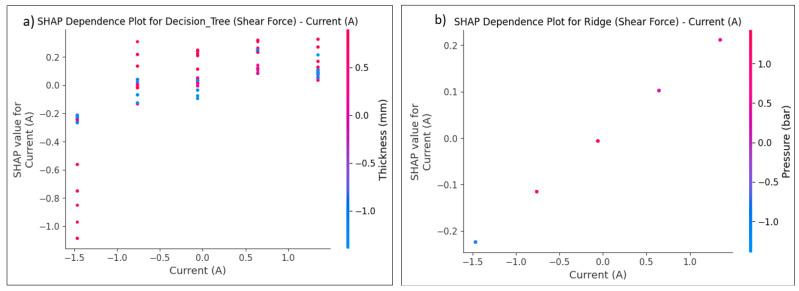
SHAP dependence plot of shear force for different algorithms: (**a**) Decision tree, (**b**) Ridge.

**Figure 30:**
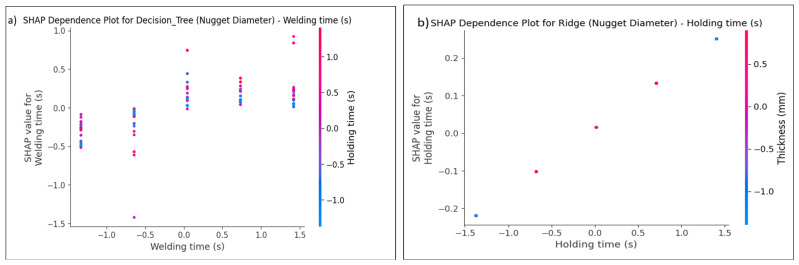
SHAP dependence plot of nugget diameter for different algorithms: (**a**) Decision tree, (**b**) Ridge.

The trends in the SHAP dependence plot are used for interpretation, which means that the plot determines whether the feature–SHAP value connection is linear or non-linear. A straight line in the graphic represents a feature with a linear influence on the model’s prediction. As seen in Figure 29b and Figure 30b, the SHAP values exhibit a consistent tendency of increasing or decreasing with changes to the feature values. This pattern was observed with shear force and nugget diameter on the Ridge model. In contrast, a non-linear shift in the feature’s impact on the prediction is shown by a curved or otherwise complicated pattern. For instance, at one moment, a little increase to the feature could make an enormous impact, while at another point, a larger rise might make less of an impact. As seen in Figure 29a and Figure 30a, this pattern emerged during the testing of the shear force and nugget diameter using the Decision tree model. Another way to evaluate for feature interactions in a dependent plot is by observing the colour of the dots. A strong interaction between two features is shown by a drastic shift in dot colour across the X-axis. This finding suggests that the main feature’s influence on the model’s prediction relies on the value of an additional feature. Based on the noticeable changes in the dot colour on the X-axis, as seen in Figure 29a and Figure 30a, it was determined that there was a strong feature interaction between sheet thickness and current for shear force, and between holding time and welding time for nugget diameter using the decision tree algorithm. In Figure 29b and Figure 30b, it can be seen that the feature interaction between shear force and current was poor, and between nugget diameter and sheet thickness and holding time when using the Ridge model. This is indicated by the fact that the colours varied across the X-axis.

### 4.4. Mathematical Equations

Analytical equations for the prediction of shear force and nugget diameter of resistance spot-welded joints were extracted from the best ANN model in order to provide a practical, efficient, and easy alternative approach to calculating shear force and nugget diameter, rather than constructing, running, and evaluating a new ANN model on a periodic basis. The result was an innovative approach, as equations may be used for the direct prediction of either the shear force or the nugget diameter by simply inserting the RSW process parameters. As a result, the best-performing ANN network’s constant weights and biases were used to formulate mathematical equations for determining the shear force and nugget diameter. Layer weight (LW) separates the hidden layer from the outputs, whereas input weight (IW) separates the hidden layer from the input parameters. There was also a bias of b1 or b2 applied to each layer. Table 17 lists the optimal ANN models with respect to the shear forces yielded (IW), (LW), b1, and b2.

(25) $$logsig\left(n\right)=\frac{1}{1+e^{-n}}$$

(26) $$y=f\left(net\right)=\left(\sum_{i=1}^{n}w_{i}x+b\right)$$

Since *y* represents the output and the output in this case is the shear force, Equation (31) becomes as follows:

(27) $$\mathrm{Shear}\;\mathrm{force}=f\left(net\right)=\left(\sum_{i=1}^{n}w_{i}x+b\right)$$

Since the $f\left(net\right)=Logsig\;(n)$, the following equations can be derived:

(28) Shear force=b2+LW × Logsig (b1+IW × x)

(29) $$\mathrm{Shear}\;\mathrm{force}=b2+\mathrm{LW}\;\times \;\frac{1}{1+exp\;(-(b1+IW\times x)}$$

Since the inputs (*x*) represent welding current, pressure, welding time, squeeze time, holding time, pulse welding, and sheet thickness, the equation becomes the following:

(30) $$\mathrm{Shear}\;\mathrm{force}=1.0742+\mathrm{LW}\;\times \;\;\frac{1}{\begin{array}{c} 1+\exp\;(-(b1+IW\times x\;(\mathrm{welding}\;\mathrm{current}\\ \;\;\;\;\;\;\;\;\;\;\;\;\;\;\;\;\;\;\;\;\;\;\;\;\;\;\;\;\;\;\;\;\;\;\;\;\;\;\;\;\;\;\;\;\;\;\mathrm{welding}\;\mathrm{time}\\ \;\;\;\;\;\;\;\;\;\;\;\;\;\;\;\;\;\;\;\;\;\;\;\;\;\;\;\;\;\;\;\;\;\;\;\;\;\;\;\mathrm{pressure}\\ \;\;\;\;\;\;\;\;\;\;\;\;\;\;\;\;\;\;\;\;\;\;\;\;\;\;\;\;\;\;\;\;\;\;\;\;\;\;\;\;\;\;\;\;\;\;\mathrm{holding}\;\mathrm{time}\\ \;\;\;\;\;\;\;\;\;\;\;\;\;\;\;\;\;\;\;\;\;\;\;\;\;\;\;\;\;\;\;\;\;\;\;\;\;\;\;\;\;\;\;\;\;\;\mathrm{squeeze}\;\mathrm{time}\\ \;\;\;\;\;\;\;\;\;\;\;\;\;\;\;\;\;\;\;\;\;\;\;\;\;\;\;\;\;\;\;\;\;\;\;\;\;\;\;\;\;\;\;\;\;\;\;\mathrm{pulse}\;\mathrm{welding}\\ \;\;\;\;\;\;\;\;\;\;\;\;\;\;\;\;\;\;\;\;\;\;\;\;\;\;\;\;\;\;\;\;\;\;\;\;\;\;\;\;\;\;\;\;\;\;\;\;\;\;\mathrm{sheet}\;\mathrm{thickness}) \end{array}}$$

where b1, IW, and LW take values shown in Table 17.

For formulating the nugget diameter equation, the following should be applied:

(31) $$tansig\left(n\right)=\frac{2}{1+e^{-2n}}-1$$

(32) $$y=f\left(net\right)=\left(\sum_{i=1}^{n}w_{i}x+b\right)$$

Since *y* represents the output and the output in this paper is the shear force, Equation (33) becomes as follows:

(33) $$\mathrm{Nugget}\;\mathrm{diameter}=f\left(net\right)=\left(\sum_{i=1}^{n}w_{i}x+b\right)$$

When applying the same steps when deriving the shear force equation, the nugget diameter equation will be the following:

(34) $$\mathrm{Nugget}\;\mathrm{diameter}=0.13238+\mathrm{LW}\;\times \;\;\frac{2}{\begin{array}{c} 1+\exp\;(-2(b1+IW\times x\;(\mathrm{welding}\;\mathrm{current}\\ \;\;\;\;\;\;\;\;\;\;\;\;\;\;\;\;\;\;\;\;\;\;\;\;\;\;\;\;\;\;\;\;\;\;\;\;\;\;\;\;\;\;\;\;\;\;\mathrm{welding}\;\mathrm{time}\\ \;\;\;\;\;\;\;\;\;\;\;\;\;\;\;\;\;\;\;\;\;\;\;\;\;\;\;\;\;\;\;\;\;\;\;\;\;\;\;\mathrm{pressure}\\ \;\;\;\;\;\;\;\;\;\;\;\;\;\;\;\;\;\;\;\;\;\;\;\;\;\;\;\;\;\;\;\;\;\;\;\;\;\;\;\;\;\;\;\;\;\;\mathrm{holding}\;\mathrm{time}\\ \;\;\;\;\;\;\;\;\;\;\;\;\;\;\;\;\;\;\;\;\;\;\;\;\;\;\;\;\;\;\;\;\;\;\;\;\;\;\;\;\;\;\;\;\;\;\mathrm{squeeze}\;\mathrm{time}\\ \;\;\;\;\;\;\;\;\;\;\;\;\;\;\;\;\;\;\;\;\;\;\;\;\;\;\;\;\;\;\;\;\;\;\;\;\;\;\;\;\;\;\;\;\;\;\;\mathrm{pulse}\;\mathrm{welding}\\ \;\;\;\;\;\;\;\;\;\;\;\;\;\;\;\;\;\;\;\;\;\;\;\;\;\;\;\;\;\;\;\;\;\;\;\;\;\;\;\;\;\;\;\;\;\;\;\;\;\;\;\;\mathrm{sheet}\;\mathrm{thickness}) \end{array}\;\;-1}$$

where b1, IW, and LW take values shown in Table 18.

## 5. Conclusions

This study presented and described different algorithms and model structures for artificial neural networks (ANNs) and machine learning (ML) models to predict shear force and nugget diameter in relation to the quality of resistance spot-welded joints made from similar and dissimilar AISI 304 austenitic stainless steel and grade 2 titanium alloy with equal and unequal thicknesses of 0.5 and 1 mm. The prediction models for shear force and nugget diameter were also evaluated using Shapley additive explanations (SHAP) values. The main purpose of the research was to find the optimal predictive model and structure by analysing the RI of the RSW parameters used in the welding process. A notable finding from this study is the importance of developing a neural network architecture using RSW parameters, including optimum training and transfer functions, to derive two equations for predicting shear force and nugget diameter from the best predictive model. The most promising results derived from the current study are as follows:

1. The most promising performance of predicting shear force in a one-output structure was achieved by way of using Trainbr as a training function and Logsig as a transfer function. On the other hand, the Trainbr training with the Purelin transfer function gave the worst prediction for the shear force one-output model.
2. Concerning the nugget diameter one-output model, it was found that the Trainrp training function with the Tansig transfer function produced the most accurate prediction, while using the Traincgp training function with the Purelin transfer function resulted in the least accurate prediction.
3. The two-output structure model confirmed that the Trainrp training function with Tansig transfer function shows optimal prediction, as in the one-output nugget diameter model. Also, using the two-output structure for predicting the nugget diameter is more favourable than the one-output nugget diameter model.
4. The Random forest ML model demonstrated the optimal prediction performance for shear force or nugget diameter in a one-output model. On the other hand, the Decision tree model gave the best prediction in the two-output model.
5. The Linear regression model produced the poorest forecast among the ML models for the shear force one-output model, while the ElasticNet model had the worst performance for the nugget diameter one-output model. When comparing the two-output models, the Lasso model performed the worst.
6. ML models such as CatBoost and Gradient-boosting had similar predictive performance in one- and two-output structures, ranking them as the second most accurate models behind the Random forest and Decision tree models.
7. The results of the relative importance (RI) analysis show that sheet thickness has the greatest impact on the shear force and nugget diameter models.
8. Pulse welding using gradient-boosting, CatBoost, and Random forest, with welding duration using decision tree, had the lowest effective parameter on shear force. The squeezing time had the least impact on the nugget diameter when Decision tree and Gradient-boosting models were used, whereas welding pressure was the least effective when CatBoost and Random forest models were used.

## 6. Recommendations for Future Work

This study addressed a number of issues that need further investigation. It is essential to investigate the effects of the studied factors on sheets of various thicknesses and materials. The microstructure of the welded joints must be well understood, utilizing methods such as scanning electron microscopy (SEM), transmission electron microscopy (TEM), or X-ray diffraction (XRD). Additionally, in order to verify and validate the derived equations, several RSW scenarios should be examined with varying parameter values.

## Figures and Tables

**Table 1:** Dimensions of the RSW specimens.

Thickness (mm)	Length (mm)	Width (mm)	Overlap Region (mm)
0.5	76	16	16
1	100	25	25

**Table 2:** Mechanical properties of AISI 304 and Ti grade 2.

Property	Tensile Strength (MPa)	Yield Strength (MPa)	Poisson’s Ratio	Young’s Modulus (GPa)	Elongation (%)
AISI 304	505	215	0.29	193	40
Ti grade 2	428	318	0.37	113	28

**Table 3:** Chemical composition of AISI 304 and Ti grade 2.

Element wt.%	C	Mn	P	S	Si	Cr	Ni	Cu	Mo	O	N	H	Ti	Fe
AISI 304	0.08	2.0	0.04	0.03	1.0	18	8	0.75	0.75	-	-	-	-	Bal.
Ti grade 2	0.0055	-	-	-	-	-	-	-	-	0.11	0.0067	0.001	Bal.	0.062

**Table 4:** RSW experimental parameters according to the design of experiment (DOE).

Trial No.	Welding Current (A)	Pressure (Bar)	Welding Time (s)	Squeeze Time (s)	Holding Time (s)	Pulse Welding (-)
1	5000	2.0	0.6	0.6	0.50	1
2	5000	3.5	0.8	0.8	0.75	2
3	5000	5.0	1.0	1	1.00	3
4	5000	6.5	1.2	1.2	1.25	4
5	5000	8.0	1.4	1.4	1.50	5
6	5500	2.0	0.8	1.0	1.25	5
7	5500	3.5	1.0	1.2	1.50	1
8	5500	5.0	1.2	1.4	0.50	2
9	5500	6.5	1.4	0.6	0.75	3
10	5500	8.0	0.6	0.8	1.00	4
11	6000	2.0	1.0	1.4	0.75	4
12	6000	3.5	1.2	0.6	1.00	5
13	6000	5.0	1.4	0.8	1.25	1
14	6000	6.5	0.6	1.0	1.50	2
15	6000	8.0	0.8	1.2	0.50	3
16	6500	2.0	1.2	0.8	1.50	3
17	6500	3.5	1.4	1.0	0.50	4
18	6500	5.0	0.6	1.2	0.75	5
19	6500	6.5	0.8	1.4	1.00	1
20	6500	8.0	1.0	0.6	1.25	2
21	7000	2.0	1.4	1.2	1.00	2
22	7000	3.5	0.6	1.4	1.25	3
23	7000	5.0	0.8	0.6	1.50	4
24	7000	6.5	1.0	0.8	0.50	5
25	7000	8.0	1.2	1.0	0.75	1

**Table 5:** Training functions used in multilayer perceptron (MLP).

Algorithm	Description
Trainlm	Levenberg–Marquardt
Trainscg	Scaled conjugate gradient
Traincgf	Conjugate gradient with Fletcher–Reeves updates
Traincgb	Conjugate gradient with Powell–Beale restarts
Traincgp	Conjugate gradient with Polak–Ribiere updates
Trainbfg	BFGS Quasi-Newton
Trainrp	Resilient backpropagation
Trainbr	Bayesian regularization backpropagation
Trainoss	One step secant
Traingd	Gradient descent backpropagation
Traingdm	Gradient descent with momentum
Traingda	Gradient descent with adaptive learning rate
Traingdx	Gradient descent momentum and an adaptive learning rate

**Table 6:** Shear force (kN) results for all RSW cases.

No.	Case A	Case B	Case C	Case D	Case G	Case H	Case E	Case F	Case I	Case J
1	4.423	2.422	3.665	5.656	2.575	3.546	0.899	1.898	1.138	1.528
2	4.052	2.317	3.916	6.788	3.26	3.459	1.025	0.666	1.107	1.463
3	4.918	3.352	3.412	6.201	2.736	3.578	1.216	0.755	0.818	1.166
4	4.737	3.687	3.96	8.583	3.139	3.606	1.261	0.849	0.703	1.554
5	4.315	4.234	4.116	6.857	3.153	3.512	1.14	0.866	0.953	1.761
6	4.713	4.086	4.421	7.358	2.662	3.443	0.973	0.861	0.68	0.835
7	4.555	3.215	4.121	5.902	2.39	2.926	1.04	1.37	1.037	1.091
8	3.958	2.895	2.916	7.71	2.596	3.376	1.617	2.053	1.171	1.515
9	4.455	3.125	3.058	7.914	2.693	3.125	0.857	1.631	0.966	1.749
10	4.618	2.552	3.896	8.152	2.447	3.25	0.898	1.073	0.685	1.188
11	4.6	2.43	3.492	8.6	2.583	3.611	1.545	0.724	0.611	0.888
12	4.239	2.591	3.969	7.646	2.401	2.948	1.455	0.784	0.779	1.623
13	4.198	2.965	3.207	7.641	2.547	3.226	1.402	2.589	0.649	1.851
14	3.861	2.36	3.918	8.279	2.609	3.297	2.183	1.055	1.032	1.09
15	4.171	2.191	3.161	5.763	2.648	3.027	0.769	1.129	1.407	1.483
16	4.271	3.76	3.343	8.05	2.594	3.756	0.94	1.17	0.585	1.072
17	4.671	3.511	3.404	8.107	2.688	2.812	0.735	0.684	0.866	1.428
18	4.558	2.789	3.57	7.299	2.821	3.506	0.59	0.476	0.723	0.909
19	4.406	2.504	3.785	5.707	2.22	2.708	1.376	2.031	0.514	1.796
20	4.141	2.721	3.402	7.33	3.226	3.869	1.836	1.21	1.708	1.841
21	5.028	3.017	4.149	8.267	3.099	3.997	0.528	0.769	1.282	1.117
22	5.009	3.031	4.165	7.346	2.746	4.031	0.564	0.882	0.628	1.097
23	4.536	2.852	4.04	6.997	2.886	3.541	0.968	0.721	1.463	1.383
24	5.106	3.261	3.741	7.945	3.298	3.439	1.953	0.66	0.892	1.265
25	4.715	3.149	2.155	8.141	2.693	3.401	1.393	1.861	1.163	1.77

**Table 7:** Nugget diameter (mm) results for all RSW cases.

No.	Case A	Case B	Case C	Case D	Case G	Case H	Case E	Case F	Case I	Case J
1	5.4	3.4	4.2	3.9	3.2	4.1	5.1	4.8	4.3	5.5
2	4.9	3.1	4.9	4.1	3.5	5	5.8	3.4	4.1	5.3
3	5.6	4.8	5	4.3	3.9	4.2	6.7	3.9	3.5	4.2
4	5.7	5.6	4.1	5.2	3.4	4.8	4.1	4.3	3.2	5.6
5	6.5	3.7	4.5	4.2	3.8	3.8	4.4	4.8	5.1	6.3
6	6.4	4	5.2	4.1	3.7	3.1	5.5	4.4	3.3	3.2
7	5.3	4.3	5.8	3.9	2.5	3.7	4.9	5.1	5.6	3.9
8	5.2	3.1	3.7	5.6	3.2	3.7	5.1	5.6	5.5	5.4
9	5	3.9	3.7	4.5	3.6	3.5	4.8	5.4	5.1	6.3
10	5.7	3.4	5.1	4.9	2.9	3.9	5.1	5.5	4.4	4.3
11	5.8	4.3	4.4	5.4	3.4	4.6	4.7	3.7	3.9	3.2
12	4.3	4.3	5.9	5.1	3.3	3.2	5.2	4.1	4.9	5.8
13	5.2	2.8	5.7	5.1	3.2	3.5	5.9	5.8	4.1	6.3
14	4.6	3.2	4	5.3	3.4	4.4	6.7	5.4	4.6	3.9
15	3.4	4.3	3.8	3.9	3.4	3.7	4.4	5.3	5.1	5.3
16	4.4	3.7	4.4	5.3	4.1	4.5	5.3	5.1	3.7	3.8
17	5.5	3.1	3.4	5.4	3.3	3	4.2	3.5	4.5	5.2
18	5.1	3	4.5	4.8	3.6	4.4	3.4	3.4	4.3	3.3
19	3.8	3.1	3.5	4.1	3	2.9	5.8	5.5	3.2	6.4
20	3.9	3.1	5.6	4.2	4.2	4.7	6.4	4.1	4.9	6.5
21	5.2	4.1	5	4.1	4.4	5.5	3.3	3.9	4.8	4.1
22	5.3	3.8	4.9	4.6	2.9	4.8	3.2	4.2	3.1	3.9
23	5.5	4.4	4.6	4.6	3.9	4.9	5.5	3.7	5.3	5.1
24	4.1	4.3	4.7	4.4	4.1	4.4	6.1	3.4	4.7	4.6
25	5.5	3.3	4	5.3	3.3	4.8	4.9	4.6	4.4	6.1

**Table 8:** Validation metrics for predicting the shear force (one output) using different training and transfer functions.

Training Function	Transfer Function	MSE	RMSE	ME	MAE
Trainlm	Tansig	0.06793	0.260639	−0.033483	0.157391
	Logsig	0.090664	0.301104	0.026366	0.209616
	Purelin	0.172688	0.415557	0.029422	0.298463
Trainscg	Tansig	0.12782	0.357525	0.028004	0.273241
	Logsig	0.14905	0.386071	0.077823	0.262628
	Purelin	0.15105	0.388652	−0.000208	0.285034
Traincgf	Tansig	0.14466	0.380354	0.01164	0.284822
	Logsig	0.15323	0.391451	0.015834	0.297386
	Purelin	0.21720	0.466050	−0.028463	0.316804
Traincgb	Tansig	0.10808	0.328763	0.022031	0.238873
	Logsig	0.12264	0.350197	0.009169	0.269599
	Purelin	0.17095	0.413460	0.018994	0.305172
Traincgp	Tansig	0.17081	0.413286	−0.019718	0.301338
	Logsig	0.17220	0.414971	0.028786	0.323124
	Purelin	0.27833	0.527567	−0.002766	0.358982
Trainbfg	Tansig	0.12089	0.347699	0.025047	0.256489
	Logsig	0.16436	0.405419	0.012246	0.313081
	Purelin	0.20635	0.454259	−0.049976	0.337784
Trainrp	Tansig	0.08369	0.289292	−0.001287	0.205665
	Logsig	0.14519	0.381038	−0.006857	0.295660
	Purelin	0.19176	0.437899	0.037569	0.301395
**Trainbr**	Tansig	0.08044	0.283611	−0.025353	0.184352
	**Logsig**	**0.06487**	**0.254687**	**0.009119**	**0.153859**
	Purelin	0.20094	0.448265	−0.018866	0.351234
Trainoss	Tansig	0.10454	0.323323	0.004677	0.211837
	Logsig	0.12677	0.356052	0.000531	0.262159
	Purelin	0.17178	0.414462	0.028899	0.305966
Traingd	Tansig	0.23516	0.484929	0.020264	0.385079
	Logsig	0.39400	0.627694	0.115261	0.476218
	Purelin	0.24842	0.498414	−0.037826	0.374744
Traingdm	Tansig	0.22535	0.474709	−0.049775	0.367649
	Logsig	0.38980	0.624339	−0.098185	0.475651
	Purelin	0.53432	0.730975	−0.197698	0.565675
Traingda	Tansig	0.18809	0.433698	−0.053362	0.337193
	Logsig	0.27932	0.528506	0.016194	0.395167
	Purelin	0.35699	0.597490	−0.003164	0.467038
Traingdx	Tansig	0.21703	0.465861	−0.009815	0.358042
	Logsig	0.30896	0.555844	0.038243	0.428516
	Purelin	0.58793	0.766763	−0.103123	0.621736

**Table 9:** The evaluation metrics for checking the accuracy of the ML models for predicting shear force.

	MSE	RMSE	ME	MAE
Linear regression model	1.543320	1.242304	0.009343	0.938112
Decision tree model	0.811960	0.901088	0.008802	0.630072
Support vector machine (SVM) model	1.293762	1.137437	−0.365242	0.615556
K-Nearest neighbour (KNN) model	0.834705	0.913622	−0.012521	0.630996
Gradient-boosting model	0.811496	0.900831	0.008887	0.630058
CatBoost model	0.811960	0.901088	0.008802	0.630072
**Random forest model**	**0.** **810029**	**0.** **900016**	**0.** **007676**	**0.** **629485**
Lasso model	1.538041	1.240178	0.007406	0.931956
Ridge model	1.543218	1.242263	0.009340	0.938037
ElasticNet model	1.540389	1.241124	0.008338	0.933144

**Table 10:** The optimized hyperparameters of all ML models for predicting the shear force.

Decision tree model	maximum depth: 10			minimum samples split: 2		
SVM model	C: 10			Kernel function: rbf		
KNN model	n neighbours: 10					
Gradient-boosting model	learning rate: 0.2			n estimators: 200		
CatBoost model	depth: 10		iterations: 200		learning rate: 0.2	
Random forest model	maximum depth: 20			n estimators: 200		
Lasso model	alpha: 0.01					
Ridge model	alpha: 0.1					
ElasticNet model	alpha: 0.01			L1 ratio: 0.2		

**Table 11:** Validation metrics for predicting the nugget diameter (one output) using different training and transfer functions.

Training Function	Transfer Function	MSE	RMSE	ME	MAE
Trainlm	Tansig	0.506392	0.711612	−0.078246	0.453143
	Logsig	0.379215	0.615805	−0.061584	0.404218
	Purelin	0.822334	0.906826	−0.125987	0.587719
Trainscg	Tansig	0.293417	0.541681	−0.045550	0.411281
	Logsig	0.520117	0.721191	−0.101974	0.523837
	Purelin	0.757153	0.870145	−0.178508	0.588463
Traincgf	Tansig	0.751951	0.867151	−0.215768	0.672189
	Logsig	0.815953	0.903301	−0.218086	0.581322
	Purelin	0.913079	0.955552	−0.193851	0.672752
Traincgb	Tansig	0.467319	0.683607	−0.087055	0.411779
	Logsig	0.522564	0.722886	−0.071347	0.475271
	Purelin	1.0569	1.028060	−0.290122	0.663997
Traincgp	Tansig	0.412141	0.641982	−0.132151	0.490081
	Logsig	0.483414	0.695279	−0.095784	0.487522
	Purelin	1.462530	1.209351	−0.241879	0.782342
Trainbfg	Tansig	0.281923	0.530964	−0.025196	0.374477
	Logsig	0.546575	0.739307	0.012142	0.559824
	Purelin	0.932745	0.965787	−0.199217	0.609063
**Trainrp**	**Tansig**	**0.215283**	**0.463986**	**−0.034052**	**0.360424**
	Logsig	0.476815	0.690518	0.061179	0.400357
	Purelin	1.102394	1.049949	−0.018302	0.780857
Trainbr	Tansig	0.263847	0.513660	−0.027536	0.273392
	Logsig	0.650645	0.806625	−0.039165	0.304105
	Purelin	0.846453	0.920029	−0.242226	0.617036
Trainoss	Tansig	0.394918	0.628427	−0.028252	0.361986
	Logsig	0.544884	0.738162	−0.079294	0.566448
	Purelin	0.933619	0.966239	−0.216209	0.640571
Traingd	Tansig	0.716968	0.846739	−0.055161	0.603499
	Logsig	0.750115	0.866092	−0.051817	0.651059
	Purelin	0.968971	0.984363	−0.025073	0.704038
Traingdm	Tansig	0.796356	0.892388	0.142323	0.678030
	Logsig	0.799563	0.894183	−0.070841	0.685069
	Purelin	1.119147	1.057898	−0.093248	0.769149
Traingda	Tansig	0.700764	0.837116	−0.146274	0.603017
	Logsig	0.726987	0.852637	−0.043993	0.614792
	Purelin	1.210560	1.100255	−0.190857	0.712195
Traingdx	Tansig	0.705925	0.840193	0.080059	0.636371
	Logsig	0.629331	0.793304	−0.066063	0.537996
	Purelin	1.213467	1.101575	−0.070494	0.779249

**Table 12:** The evaluation metrics for checking the accuracy of the ML models for predicting nugget diameter.

	MSE	RMSE	ME	MAE
Linear regression model	0.865809	0.930489	−0.035236	0.623407
Decision tree model	0.509383	0.713711	−0.045667	0.427667
Support vector machine (SVM) model	0.644345	0.802711	0.104745	0.391535
K-Nearest neighbour (KNN) model	0.513015	0.716251	−0.030267	0.432733
Gradient-boosting model	0.508880	0.713358	−0.045024	0.427618
CatBoost model	0.509383	0.713711	−0.045667	0.427667
**Random forest model**	**0.** **508039**	**0.** **712768**	**−0.** **045506**	**0.** **427442**
Lasso model	0.868458	0.931911	−0.033639	0.620585
Ridge model	0.865827	0.930498	−0.035206	0.623276
ElasticNet model	0.869719	0.932587	−0.033409	0.620323

**Table 13:** The optimized hyperparameters of all ML models for predicting the nugget diameter.

Decision tree model	maximum depth: none			minimum samples split: 2		
SVM model	C: 10			Kernel function: rbf		
KNN model	n neighbours: 10					
Gradient-boosting model	learning rate: 0.2			n estimators: 200		
CatBoost model	depth: 10		iterations: 200		learning rate: 0.2	
Random forest model	maximum depth: 10			n estimators: 200		
Lasso model	alpha: 0.01					
Ridge model	alpha: 0.1					
ElasticNet model	alpha: 0.01			L1 ratio: 0.2		

**Table 14:** Validation metrics for predicting the shear force and nugget diameter (two outputs) using different training and transfer functions.

Training Function	Transfer Function	MSE	RMSE	ME	MAE
Trainlm	Tansig	0.291830	0.540213	−0.070159	0.354481
	Logsig	0.208360	0.456465	−0.031935	0.321598
	Purelin	0.443644	0.666066	−0.054964	0.429347
Trainscg	Tansig	0.318879	0.564694	−0.027799	0.413714
	Logsig	0.465147	0.682017	−0.115274	0.441985
	Purelin	0.776054	0.880939	−0.122462	0.553137
Traincgf	Tansig	0.489873	0.489873	−0.062532	0.379675
	Logsig	0.611085	0.611085	−0.046564	0.467321
	Purelin	0.719394	0.719394	−0.032411	0.433128
Traincgb	Tansig	0.236814	0.486635	0.002193	0.357157
	Logsig	0.530881	0.728616	−0.088304	0.478291
	Purelin	0.800434	0.894670	−0.129002	0.546305
Traincgp	Tansig	0.388944	0.623654	−0.091575	0.369346
	Logsig	0.424308	0.651389	0.035326	0.451587
	Purelin	0.581166	0.762343	−0.120863	0.454141
Trainbfg	Tansig	0.207497	0.455519	−0.003774	0.323576
	Logsig	0.351263	0.592675	−0.001942	0.433285
	Purelin	0.691499	0.762343	−0.199218	0.609063
**Trainrp**	**Tansig**	**0.040719**	**0.438666**	**−0.035444**	**0.318671**
	Logsig	0.042783	0.490480	−0.046581	0.348106
	Purelin	0.112521	0.767294	−0.132692	0.483787
Trainbr	Tansig	0.263847	0.409218	0.005178	0.239807
	Logsig	0.06487	0.428889	−0.032972	0.223460
	Purelin	0.846453	0.752678	0.002132	0.002132
Trainoss	Tansig	0.552631	0.743392	−0.131855	0.499736
	Logsig	0.610970	0.781646	−0.099502	0.522669
	Purelin	0.647130	0.804444	−0.155771	0.521275
Traingd	Tansig	0.524816	0.724442	−0.116380	0.534945
	Logsig	0.682575	0.826181	−0.074603	0.611539
	Purelin	0.945456	0.972346	−0.268925	0.732328
Traingdm	Tansig	0.625232	0.790716	−0.070112	0.572119
	Logsig	0.912273	0.955130	−0.196272	0.646151
	Purelin	1.078484	1.038501	−0.008129	0.778287
Traingda	Tansig	0.632192	0.795105	0.004059	0.569958
	Logsig	0.670544	0.818868	−0.030598	0.584938
	Purelin	1.166745	1.080159	−0.166737	0.796657
Traingdx	Tansig	0.652514	0.807783	−0.060835	0.599373
	Logsig	0.792063	0.889979	0.075344	0.624277
	Purelin	0.904848	0.951235	−0.038455	0.685373

**Table 15:** The evaluation metrics for checking the accuracy of the ML models for predicting shear force and nugget diameter.

	MSE	RMSE	ME	MAE
Linear regression model	1.192079	1.091824	1.657 × 10^−16^	0.713365
Decision tree model	0.617400	0.785748	−4.736 × 10^−17^	0.490667
Support vector machine (SVM) model	1.057539	1.028367	−0.133193	0.473976
K-Nearest neighbour (KNN) model	0.766689	0.875608	−0.018063	0.432733
Gradient-boosting model	0.682354	0.826047	−0.021342	0.453671
CatBoost model	0.711931	0.843760	−0.223135	0.446871
Random forest model	0.633338	0.795826	−0.014979	0.500870
Lasso model	1.226762	1.107593	−0.016568	0.766391
Ridge model	1.192963	1.092229	1.657 × 10^−16^	0.714742
ElasticNet model	1.210474	1.100215	1.894 × 10^−16^	0.727429

**Table 16:** The optimized hyperparameters of all ML models for predicting the shear force and nugget diameter.

Decision tree model	maximum depth: none			minimum samples split: 2		
SVM model	C: 0.1			Kernel function: rbf		
KNN model	n neighbours: 5					
Gradient-boosting model	learning rate: 0.01			n estimators: 50		
CatBoost model	depth: 10		iterations: 200		learning rate: 0.2	
Random forest model	maximum depth: 10			n estimators: 200		
Lasso model	alpha: 0.1					
Ridge model	alpha: 10					
ElasticNet model	alpha: 0.1			L1 ratio: 0.5		

**Table 17:** Weights and biases for predicting shear force with Trainbr and Logsig.

IW							b1		
3.2261	−0.37281	−0.30864	−1.251	0.19497	−0.20289	1.8982	−4.1632		
−2.2739	0.23639	−1.8274	0.20704	−0.43047	−2.1387	0.70117	3.9376		
−1.4778	−0.88999	2.2586	−1.4214	0.03182	−2.4128	0.69492	1.8717		
−1.5203	1.9471	0.0338	0.65418	−1.6979	3.0219	2.4608	−0.28168		
−1.9255	−0.66302	−1.1498	−0.53464	2.5951	−1.9784	−0.19671	0.63865		
−2.3571	0.76051	2.1638	−0.91707	−0.17238	−1.3801	−1.3242	−0.68471		
1.4875	0.2885	−1.7032	2.3832	0.59165	1.4149	2.4776	0.89504		
−0.040707	1.3755	−0.91084	−2.0045	−0.83152	2.5048	−1.0449	1.6603		
1.3249	−1.1102	−1.1674	−1.9283	−1.486	−0.83973	−3.7849	3.6758		
1.3881	0.8473	−0.81029	2.529	−0.16841	2.5019	−0.61914	3.8844		
**LW**									
1.0497	1.2008	0.15206	0.37408	−0.24062	−1.4801	−2.0563	−0.10582	1.5763	0.30685

**Table 18:** Weights and biases for predicting nugget diameter with Trainrp and Tansig.

IW							b1		
0.65839	0.16521	−2.1476	0.16065	−0.14811	1.8745	−1.7941	−1.4317		
0.028815	−0.72185	0.11913	0.14259	1.5548	1.4589	−0.28386	−1.3459		
−0.19947	0.61547	0.32509	0.49204	1.0999	0.20842	−0.99804	0.94429		
−2.3805	−0.48447	0.33117	2.2955	−0.23516	−0.35453	−0.45699	0.88384		
−0.94844	1.7365	−0.63679	−0.14787	−0.24801	−0.85397	−0.72771	−0.068722		
−0.96197	0.041092	−1.5077	−0.99344	−0.36625	−0.23195	−1.0437	−0.20019		
1.3136	1.3537	−0.74685	−0.061567	−1.91	−1.094	−1.1703	0.57311		
0.14707	−0.4577	−0.11977	−0.5746	−0.98779	0.90052	0.36406	−1.207		
0.34539	0.15692	0.21935	−0.35343	0.3723	0.9375	−0.46254	1.8719		
−0.74918	0.91938	0.27981	1.2491	0.94216	1.1141	0.34248	−2.2177		
**LW**									
0.039962	−0.3472	−0.24808	−0.14526	−0.33405	0.20147	−0.19826	−0.34985	0.36349	0.42314

## Data Availability

The original contributions presented in the study are included in the article, further inquiries can be directed to the corresponding author/s.
